# The connection between autophagy and Alzheimer’s disease

**DOI:** 10.1007/s00011-025-02118-0

**Published:** 2025-10-22

**Authors:** Nechushtai Lior, Dahan Chen, Frenkel Dan, Pinkas-Kramarski Ronit

**Affiliations:** 1 Department of Neurobiology, School of Neurobiology, Biochemistry and Biophysics, Tel-Aviv University, 69978 Ramat-Aviv, Israel; 2 Sagol School of Neuroscience, Tel-Aviv University, 69978 Ramat-Aviv, Israel

**Keywords:** Alzheimer’s disease (AD), Amyloid β, Apolipoprotein E4 (APOE4), Autophagy

## Abstract

Alzheimer’s disease (AD) is the most prevalent neurodegenerative disease associated with accumulation of amyloid beta peptides and intracellular neurofibrillary tangles formed by hyperphosphorylated Tau. Autophagy, an evolutionarily conserved process of self-degradation and turnover of cellular constituents, is important for normal cell growth but may be defective in diseases. A growing body of data implies that autophagy strongly affects AD pathogenesis. Autophagy mediates degradation of damaged organelles and proteins as well as neurotoxic aggregates, by regulating their clearance. Thus, impaired autophagy may account for the accumulation of protein aggregates. Since AD is characterized by neuroinflammation, impaired mitochondrial and lysosomal functions, and the accumulation of protein aggregates, the roles of autophagy/mitophagy in Alzheimer’s will be extensively evaluated. In the current review, we will discuss the connection between autophagy/mitophagy and Alzheimer’s. It seems that Alzheimer-related proteins such as APOE4, TREM2, PSEN1/2, APP and Tau can regulate autophagy. In turn, depending on the cellular system and animal model, autophagy regulating proteins such as Atg7, BECN1, GSK3B, MAP1LC3B, SQSTM1, TFEB and VCP can affect AD progression as discussed. We will also describe the effect of sex and lifestyle impact on autophagy and AD. Finally, we will describe how the current knowledge may contribute to potential therapeutic strategies.

## Introduction

Alzheimer’s disease (AD) is the most prevalent form of dementia [1] and the most common neurodegenerative disease [2, 3]. It is characterized by decline in thinking, memory and language skills, as well as personality changes and impairment in performing daily tasks [4]. Two molecular hallmarks of AD include toxic extracellular plaques of amyloid beta (Aβ) peptides and intracellular neurofibrillary tangles (NFTs) formed by hyperphosphorylated Tau [2]. Age and family history are among the risk factors for the disease. Early-onset familial AD (EOFAD) mutations are autosomal dominant and represent less than 5% of AD cases, while late onset AD (LOAD) may be sporadic and genetic. The EOFAD mutations are found mainly on Aβ precursor protein (APP), presenilin 1 (PSEN1/PS1) and presenilin 2 (PSEN2/PS2) [5, 6].

**Fig. 1:**
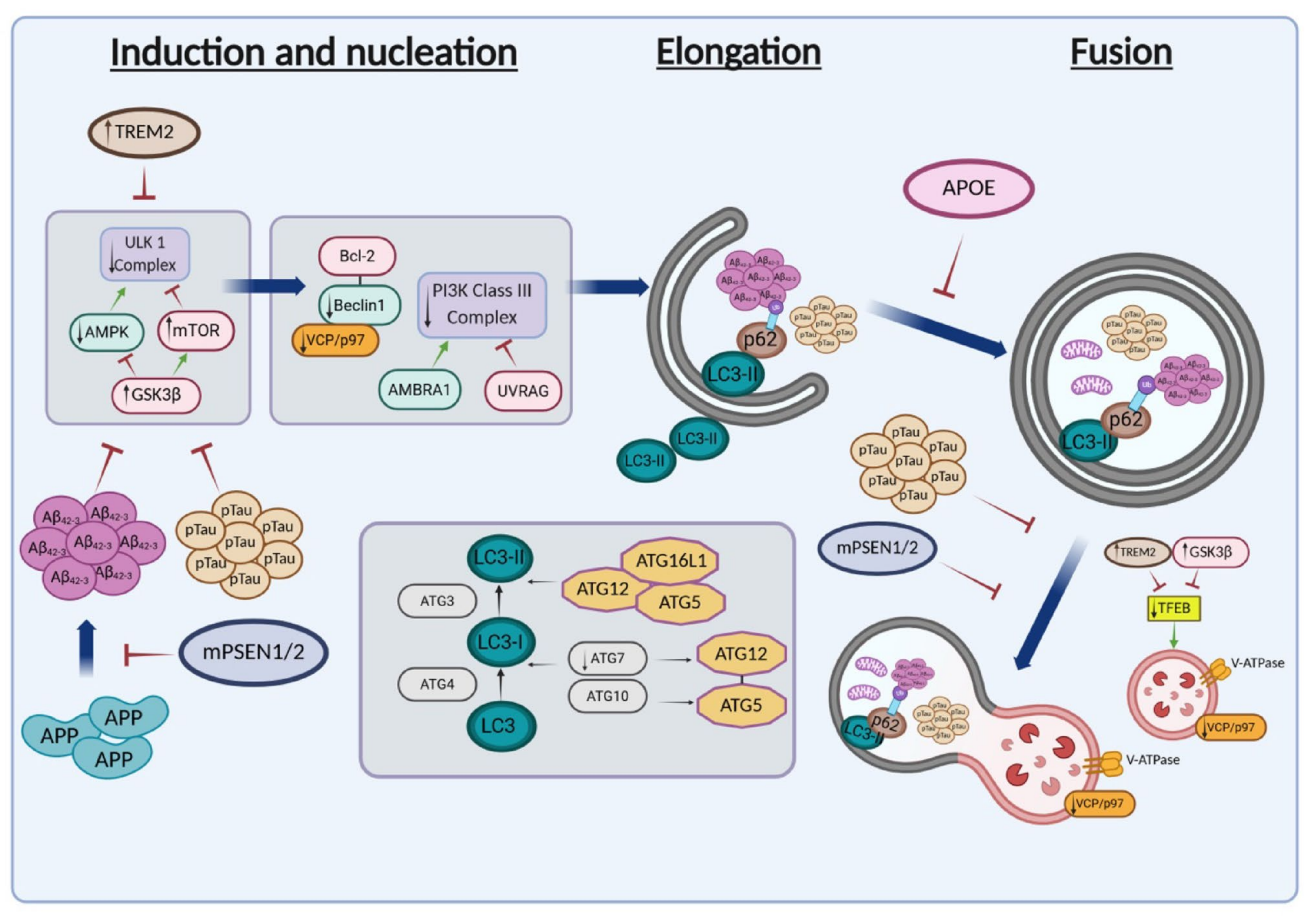
A schematic presentation of key proteins involved in autophagy regulation, alongside proteins associated with AD that may affect or be affected by autophagy. An up arrow indicates that the level of the protein is increased, whereas down arrow indicates that the level is decreased in AD

Autophagy is one of the main degradative pathways that clear damaged organelles and defective or aggregated proteins through the autophagolysosomes [7]. Under normal conditions, autophagy removes toxic proteins or organelles; however, the ability to undergo autophagy progressively declines with aging, thus can contribute to the accumulation of Aβ and Tau [8]. Impaired autophagic/lysosomal function is evident in AD. Autophagy-related gene 7 (*Atg7*) deficient mice die 28 weeks after birth and show massive neuronal loss [9]. In addition, *atg5* −/− cells demonstrated abnormal intracellular proteins accumulation, suggesting the involvement of autophagy in neurodegeneration [10]. Nixon et al. provided the first evidence for the involvement of macroautophagy in AD. They showed that autophagy vesicles (AVs) were rarely seen in neurons from brains of individuals with no neuropathology, but were frequently seen in AD brains [11]. It was suggested that autophagy may be involved in Aβ clearance [12, 13]. Also, it was shown that familial form of AD (FAD) genes such as *PSEN1* maintain lysosomal acidification and autophagy, and *PSEN1/2* mutations disrupt autophagosome–lysosome fusion and biogenesis [5, 14]. As a result, Aβ aggregates and phosphorylated Tau (pTau) can accumulate [15]. In fact, macroautophagy is thought to be the main route to remove both Aβ and Tau aggregates, and pharmacological enhancers of autophagy may reduce these aggregates [8]. Additional genes that may be involved in the pathology of AD and in autophagy are presented in Fig. 1 and Table 1. More understanding of the involvement of autophagy in AD pathology may lead to new therapeutic approaches. In the present review we will discuss the connection between autophagy and AD, as well as the relation between AD risk factors and autophagy.

**Table 1:** AD related proteins that affect autophagy, and autophagy related proteins that may affect AD

Protein	Connection to AD	Autophagy/mitophagy	References
Amyloid beta Aβ	Increase of oligomerization of soluble and insoluble Aβ	Impaired autophagy, mitophagy and Aβ clearance	[33–35, 40–42, 50]
Apolipoprotein E4 (APOE4)	A major genetic risk factor for LOAD	Impairs autophagy and mitophagy	[81–91]
Presenilin (PSEN) 1 and 2	Mutant PSEN is linked to about 75% of EFAD	Impairs autophagy and mitophagy	[96–99, 102]
Tau	Abnormal Tau phosphorylation forms neurofibrillary tangles	Impairs autophagy and Tau degradation	[54–56, 61–63, 65–67]
Triggering receptor expressed on myeloid cells 2 (TREM2)	Mutations in TREM2 are associated with increased AD risk	Loss of function impairs autophagy	[114, 116, 189, 190]
Autophagy related protein7 (Atg7)	Reduced activity in AD	Impairs autophagy and cause the accumulation of Aβ and Tau	[49, 129–132]
Beclin1	levels are reduced in AD patient	Impaired autophagy	[117, 118, 121, 122]
Glycogen synthase kinase 3β (GSK3β)	GSK3β is hyperactive in AD brains	Impairs autophagy and affects lysosome activity and Aβ degradation	[133, 134, 191]
sequestosome 1 (SQSTM1), p62	P62 levels are increased in AD	Impaired autophagy	[8, 192, 193, 194] 10/14/2025 7:19:00 AM
Transcription factor EB (TFEB)	TFEB is dysregulated in AD	Impairs autophagy and affect Aβ and Tau clearance	[76, 195, 196]
Valosin-containing protein (VCP/p97)	Decreased levels of VCP/p97 in AD	Impaired autophagy	[68, 197]

## Autophagy and neuronal stress

Autophagy is a cellular degradation process that maintains normal cell homeostasis by degrading unwanted damaged proteins and organelles [7, 16]. There are three types of autophagy: macroautophagy, microautophagy and chaperone-mediated autophagy (CMA) [16]. Several neurodegenerative disease are characterized by accumulation of protein aggregates, and autophagy plays a crucial role in their clearance [17]. Thus, impaired autophagy was suggested to contribute to neurodegenerative pathologies [17, 18]. In neurons it was suggested that autophagy has a protective effect although several studies suggest that autophagy may induce neuronal cell death [19, 20].

The process of autophagy includes several steps: initiation and nucleation, sequestration of proteins and organelles destined for degradation into a double- membrane vesicle termed autophagosome, and fusion of the autophagosome with the lysosome for degradation (Fig. 1) [21, 22]. Induction can begin with the regulation of Unc51-like autophagy activating kinase 1 (ULK1) complex by mammalian target of rapamycin (mTOR) and Adenosine-5′-monophosphate-activated protein kinase (AMPK) [23]. This process also involves ATGs such as Atg13 and Atg101. ULK1 also affects autophagy induction by activating the Class III Phosphoinositide 3 kinase (PI3K) which is regulated by Bcl2 and Beclin1, as well as UV radiation resistance-associated gene (UVRAG) and Activating molecule in Beclin1 regulated autophagy protein 1 (AMBRA1) [24]. Next, two essential ubiquitin like conjugation systems operates which include light chain 3 (LC3) lipidation and Atg5/Atg12 conjugation which are important for phagophore expansion and cargo selection [21, 25]. In the first system, Atg3, Atg4, and Atg7 mediate the conjugation of phosphatidyl-ethanolamine (PE) to Atg8/LC3. The second system involves a covalent linkage between Atg5 and Atg12, which are noncovalently associated with Atg16. This complex is required for the growth of the phagophore and is mediated by Atg7 and Atg10 [26, 27]. The adaptor Sequestosome1 (SQSTM1)/p62 mediates cargo selection by binding to LC3 and recognizing ubiquitinated proteins [22]. The fusion of the autophagosome and the lysosome to form autophagolysosomes is facilitated by regulation of UVRAG and RAB7 [22]. Dysregulation of autophagy may contribute to the pathogenesis of several neurodegenerative diseases, by impairing the removal of protein aggregates such as Aβ and Tau [28]. Impaired autophagy can be induced by excessive accumulation of Aβ and Tau resulting in less effective Aβ and tau clearance, leading to neuroinflammation. It has been shown that ATG5 or Beclin-1 gene deficiency in microglia activates NF-κB signaling, induces the generation of mitochondrial reactive oxygen species (ROS), and release of proinflammatory cytokines [29]. Knockdown of Atg7 or LC3 induces NLRP3 inflammasome activation, in microglia treated with Aβ aggregates, leading to the release of the proinflammatory cytokine IL-1β [30]. Also, increased neuroinflammation and neuronal loss in response to Aβ, were observed in Alzheimer’s transgenic mice microglia with *Atg7* knockout or Beclin1 deficiency [31]. These studies suggest that autophagy is important for regulation of neuroinflammation in AD.

## Autophagy and amyloid β

One of the pathologies of AD is the extracellular plaque deposits of Aβ peptide derived from APP [32–35]. APP undergoes processing that includes several cleavage steps mediated by α-, β-, and γ-secretases. The α-secretase cleavage leads to a non-amyloidogenic pathway that prevents the formation of Aβ, while the amyloidogenic pathway starts with β-secretase, followed by the activity of γ-secretase [35]. It has been suggested that the aggregation of Aβ into senile plaques (SP) can lead to neurotoxicity and dementia, contributing to the pathogenesis of AD, although neuronal loss has also been observed in areas lacking Aβ plaques [36]. In addition, autophagy has been suggested to be involved in Aβ secretion [37]. Sonda et al. claim that deterioration of autophagy with age leads to diffusion of Aβ oligomers and may contribute to AD development [38]. Also, it was suggested that senescence can affect autophagy and intraneuronal Aβ accumulation [39].

Several studies have suggested that autophagy enhancement contributes to a reduction in Aβ-plaque levels, as neurons degrade intracellular Aβ, and glial cells degrade extracellular Aβ through autophagy [40]. For example, according to Singh et al. autophagy enhancement by rapamycin facilitated protection against Aβ-mediated oxidative stress and neurotoxicity in SH-SY5Y cells [41], and abolished cognitive impairment while reducing Aβ levels in a mouse model of AD [42]. Rifamicin, an anti-bacterial agent, has also been demonstrated to enhance autophagy [43], to ameliorate cognitive impairments in LPS-induced AD mouse model and to reduce Aβ levels [44]. In addition, depletion of *Atg5* enhanced the accumulation of immature APP (iAPP) in response to Tetrahtdrohyperforin (IDN5706)-semisynthetic derivative of hyperforin, the active molecule in the St. John’s Wort plant (Hypericum perforatum), by slowing its degradation. This finding suggests that IDN5706 promotes degradation of iAPP by activation of Atg5-dependent autophagy [45]. The role of AMPK in autophagy-mediated Aβ clearance appears to differ between astrocytes and neurons. While mTORC1 inhibition consistently enhances autophagy and reduces Aβ levels in both cell types, AMPK activation increases Aβ secretion only in astrocytes [46, 47]. Also, a transcriptional frameshift of the ubiquitin B peptide generating the mutated version (UBB^+1^), reduces Aβ toxicity in yeast by activating autophagy [48]. According to Nilsson et al. knockdown of *atg7* leads to Aβ accumulation in the Golgi. Therefore, it was suggested that Atg7 affects the transport of Aβ from the Golgi to vesicular bodies [49]. In addition, astrocytes-specific knock down of LC3B and p62 significantly increased Aβ plaques and reduced neuronal markers and cognitive function [12]. Several gene therapy approaches aimed at targeting autophagy related genes have been explored. It was demonstrated that activation of Peroxisome proliferator activated receptor alpha (PPARA/PPARα) which is involved in fatty acid metabolism and in autophagy activation, decreased amyloid pathology and reversed memory deficits and anxiety symptoms in APP-PSEN1ΔE9 mice [50].

**Fig. 2:**
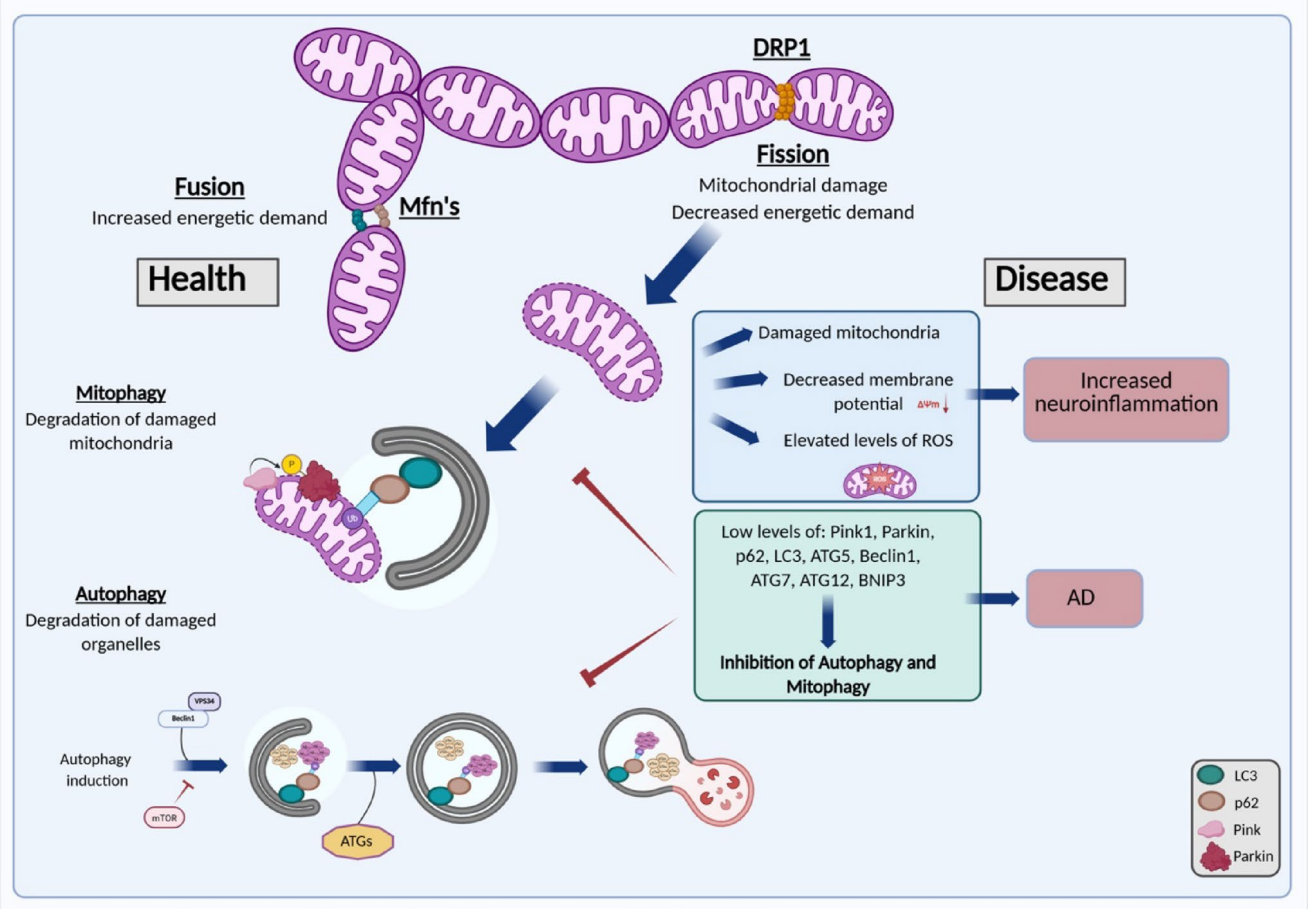
A schematic presentation describing mitochondrial dynamics, mitophagy and autophagy function under health and disease conditions. The illustration presents the balance between mitochondrial fission and fusion, the removal of damaged mitochondria by mitophagy, and the role of autophagy in maintaining cellular homeostasis. Disruption of these processes contributes to cellular dysfunction and as indicated in the scheme, it is linked to AD

Several studies have indicated the involvement of selective autophagy mechanisms such as CMA and mitophagy in AD. In APP/PS1 mice, Aβ accumulation led to mitochondrial imbalances that increased mitophagy [51]. As demonstrated in Fig. 2, mitochondrial dynamics include fission, fusion and mitophagy depending on energetic demands. Damaged or unwanted mitochondria can undergo mitophagy allowing cells to rid themselves of unwanted or damaged mitochondria. Low expression levels of autophagy regulating genes may result in inhibition of mitophagy. It has been demonstrated that agonists of Glucagon-like peptide-1 (GLP-1) and Cholecystokinin (CCK) receptors improved cognitive deficits, reduced Aβ accumulation, and alleviated mitochondrial damage in 5xFAD mice, by inducing mitophagy [52]. Dou et al. designed an Aβ oligomer binding peptide containing three CMA motifs (KFERQ), which binds to heat shock cognate 70 kDa protein (Hsc70), and demonstrated that this peptide helps Aβ oligomers to enter endosomes and lysosomes. Moreover, they showed that the peptide helps reduce Aβ oligomer levels in pluripotent stem cell (iPSC) cortical neurons derived from fibroblasts of AD patients. Additionally, primary cultured cortical neurons were protected from Aβ oligomers in the presence of the peptide [53].

## Autophagy and Tau pathology

Another AD hallmark is NFTs, which are composed of hyperphosphorylated aggregates of the microtubule‐associated protein tau (MAPT) and SP [54–56]. Tau is expressed in axons and interacts with tubulin to facilitate microtubule assembly and stability [55, 57]. It has also been suggested to promote neuronal survival via β-catenin stabilization and the regulation of organelles and biomolecules transport along axons [58]. Caballero et al. showed that following acetylation, Tau is degraded by macroautophagy and endosomal mitophagy, whereas under normal conditions, Tau is primarily degraded through CMA [59].

Pathological pTau displays altered solubility and forms filaments which reduce its ability to bind microtubules [55, 60]. Changes in soluble Tau may be affected by age-related autophagy [61]. Several studies have demonstrated that autophagy induction alleviates Tau pathology. For example, Zheng et al. showed that conventional protein kinase C (cPKC)γ reduces the levels of pTau by promoting autophagy through the AMPK/mTOR signaling pathway [62]. In addition, activation of AMPK and inhibition of mTOR through transient receptor potential vanilloid (TRPV1), enhances autophagy and the degradation of abnormally accumulated Tau. Also, blocking AMPK reduced autophagy and promote the degradation of Tau in neurons [63], and Polyethylene glycol (PEG) increased autophagy and induced Tau degradation in N_2_a cell line [64]. Nuclear Dot Protein 52 (NDP52) is an autophagic receptor, involved in the clearance of pathological pTau [65], and in autophagosome maturation. A variant of NDP52, termed NDP52^GE^, promotes autophagic degradation of pTau [65, 66]. The expression of another autophagy receptor, Optineurin (OPTN), which recognizes infected pathogens, protein aggregates and mitochondria, has also been shown to decrease the levels of pTau [67]. Inhibitor Kappa B Kinase (IKKβ) has been shown to induce autophagy and to reduce the levels of pTau in an immortalized hippocampal cell line [68]. Additionally, the expression of valosin-containing protein (VCP), an essential chaperone, enhanced autophagy flux and improved Tau degradation [68]. Rapamycin, an autophagy inducer, enhanced clearance of Tau in COS-7 cells, and decreased Tau toxicity in vivo [69]. Silva et al. demonstrated that mTOR inhibitors OSI-027, AZD2014, and AZD8055 are more potent than rapamycin in downregulating phosphorylated and insoluble Tau [70].

Traumatic brain injury (TBI), which is also a risk factor for AD, was investigated by Sweeney et al. who showed that overexpression of Bcl2 associated athanogene 3 (BAG3) in hippocampal neurons of human *tau* knock-in (hTKI) mice increased autophagy flux and reduced Tau hyperphosphorylation, synaptic dysfunction, and cognitive deficits [71]. In addition, Ji et al. showed that BAG3 interacts with the post-synaptic cytoskeleton protein synaptopodin (SYNPO), and this interaction affects Tau degradation by autophagy [72]. Also, the natural flavonoids compound, myricetin, stabilizes the interaction between Tau and Atg5, thus promoting the clearance of pTau and almost completely eliminating Tau neurotoxicity in SH-SY5Y cells expressing Tau [73]. Tau secretion is also mediated by autophagy [74]. In addition, Falcon et al. showed that Galectin-8-mediated, NDP52-dependent selective autophagy, protected against seeded Tau aggregation [75].

The truncated Tau35 leads to lipid accumulation following inhibition of transcription factor EB (TFEB) resulting in lysosomal dysfunction [76]. Ferrar et al. demonstrated that in opposed to physiological Tau, pathological Tau recognized p62 but failed to recruit Tax1-binding protein 1 (TAX1BP1), which is essential for Tau degradation [77]. Pathological Tau has also been reported to affect autophagy. Overexpression of Tau impaired the fusion of autophagosomes with lysosomes, which resulted in autophagosome accumulation [78].

## AD related genes that may affect autophagy

### Apolipoprotein E4 (APOE4)

The lipid transporter apolipoprotein E (APOE) has three isoforms: APOE2, APOE3 and APOE4 [79]. *Apoe4* is a major genetic risk factor for LOAD [79, 80]. It was shown that the presence of a single *apoe4* allele increases the risk of AD [81], and affects the accumulation of intraneuronal Aβ [82]. The APOE4 frequency in the general population is approximately 15%, but is over 60% in patients with AD [83]. APOE4 affects both Aβ and Tau pathologies. It regulates Aβ fibril formation by interacting strongly with Aβ, and leads to increasing levels of pTau [84]. Shi et al. suggested that APOE4 affects Tau pathogenesis independently of Aβ pathology [85]. Moreover, APOE4 exacerbates the senescence of hippocampal neurons and spatial cognitive impairment by downregulating acetyl-CoA levels [86], and neurons expressing APOE4 are also more prone to cell death compared to cells expressing APOE2 and APOE3 [84].

APOE4 has been suggested to mediate its effects in AD via impairment of autophagy [13, 87–90]. AD patients expressing APOE4, exhibit low mRNA levels of the two autophagic markers p62 and LC3 [87]. APOE4 expressing astrocytes and microglia show impaired autophagic flux, mitophagy function and Aβ uptake [13, 88, 91, 92], and APOE4 expressing neurons exhibited elevated levels of Aβ_42_ secretion [92]. In addition, in APOE4 carriers, the expression of Forkhead box O3A (FoxO3a), an autophagy regulator, was reduced, and led to the decrease of other autophagy mediating downstream proteins, such as Atg12, Beclin1, BNIP3 and PINK1 [93]. Moreover, Chen et al. demonstrated that APOE3 reduced amyloid deposition, enhanced microglial reactivity and protected against Aβ- induced Tau seeding [94]. In addition, APOE degradation has been suggested to be mediated by autophagy with an important role for lysosome-associated membrane protein type 2A (LAMP2A) [95].

### PSEN1/PSEN2

Mutations in the *PSEN1* and *PSEN2* genes are involved in most cases of FAD. Presenilin is essential for γ-secretase activity, a key component of the APP cleavage machinery that generates the Aβ peptide [96–99]. It was also reported that PSENs play an important role in microglia activation [100, 101]. PSENs, particularly PSEN1 are suggested to maintain the balance between endosomal/autophagic degradation and extra cellular vesicle secretion [101]. PSEN can affect autophagic function independently of its role in γ-secretase activity [102]. For example, PSEN2 mutations impair autophagy by decreasing GTPase RAB7 recruitment to autophagosomes [103]. In addition, transgenic mice expressing mutant APP and mutant PSEN1 exhibit lysosomal dysregulation in neocortical and hippocampal neurons [104]. Lee et al. showed that PSEN1 is essential for lysosomal function during autophagy, by targeting v-ATPase and affecting lysosomal acidification [105]. However, Zhang et al. suggest that PSEN1 and PSEN2 regulate lysosomal biogenesis and function not necessarily by v-ATPase [106]. Moreover, Coen et al. demonstrated that PSEN^−/−^ cells exhibit lysosomal dysfunction not as a consequence of v-ATPase dysfunction, rather as a result of alterations in lysosomal calcium storage and release [107]. According to Checler et al. PARK2 (Parkin) which acts upstream of PINK1 is regulated by a PSEN-dependent mechanism. They also proposed that PSEN1 and PSEN2 regulate autophagy and mitophagy by controlling PINK1 transcription [108].

### TREM2

One of the reasons for LOAD is associated with mutations of receptors expressed in microglia. Triggering receptor expressed on myeloid cells 2 (TREM2) is an example for these receptors and it plays a role in AD [109–112]. It recognizes phospholipids, apoptotic cells and lipoproteins [112], and is highly expressed in the hippocampus, spinal cord and white matter. It was also suggested to mediate microglia recruitment to amyloid plaques [111]. Some studies suggest that TREM2 is related to autophagy. For example, in a mouse model of TREM2 deficiency, Uland et al. showed increased number of autophagic vesicles due to impaired mTOR signaling [112]. In addition, overexpression of TREM2 in BV-2 cells alleviated inflammation by maintaining metabolic homeostasis and mitochondrial autophagy activity [113]. Another study has found that TREM2 deficiency improved microglial phagocytosis and autophagic-lysosomal activation [114], and intermittent hypoxia training (IHT) which reduces oxygen concentration in APP/PS1 mice, enhanced Aβ clearance by recycling TREM2 through VPS35 and TFEB [115]. Furthermore, soluble TREM2 that was injected into 5xFAD mice brains reduced amyloid plaques and improved memory [116].

## Autophagy related genes that may affect AD

### Beclin1

Beclin1 protein promotes autophagy and is part of the class III PI3K complex [117, 118]. It was previously shown that Beclin1 levels increase following TBI as part of the recovery mechanism [119–121]. It was also shown that changes in the levels of Beclin1 in APP transgenic mice affect extracellular amyloid pathology. Reduction of Beclin1 increased the amyloid plaques, while expression of Beclin1 by lentiviral vector reduced amyloid plaques [121]. Jeager et al. demonstrated that the changes in Beclin1 expression affect APP degradation, thus altering the levels of amyloid plaques. Also, they found less Beclin1 in brains of AD patients [122]. In aged, but not young transgenic mice expressing APP, it was shown that Beclin1 affects autophagy [123], and Saha et al. have shown that Beclin1 is involved in autophagy death program following Aβ neurotoxicity [124]. Several studies have suggested a link between Beclin1 and autophagy in neurodegeneration. For example, in brains from aged animals, the levels of palmitoyl acetyltransferase DHHC5, which regulates the palmitoylation of Beclin1, decrease and thus affect autophagy. In two models of AD, 5xFAD and PS19 that were backcrossed with *Dhhc5* knockout mice it was shown that DHHC5 deficiency impairs neurons activity and amplifies neurodegeneration by reducing autophagy [125]. In addition, acetylation of Beclin1 impairs the autophagic flux and contributes to AD pathology [126]. Beclin1 also affects AD pathology through the immune system. It impairs the recycling of TREM2 and the phagocytosis of Aβ in vitro and in vivo [127]. Also, reduction of Beclin1 alters the levels of IL-1β and IL-18, as well as inflammasome regulation, resulting in decreased microglial phagocytosis [31]. Xue et al. suggested that induction of autophagy by activation of Beclin1 before Aβ treatment can prevent neuronal death [128].

### Autophagy related gene 7 (*atg7*)

Autophagy related protein 7 (Atg7) regulates autophagy conjugation systems which promote the elongation of the autophagosomal membrane [129]. Impaired Atg7 function has been associated with neurodegeneration [130]. *Atg*7 conditional knockdown (cKO) in the forebrain of postnatal mice led to accumulation of pTau and glycogen synthase kinase 3 β (GSK3β) as well as impaired memory [131]. It was shown that in Atg7-deficient APP mice, Aβ accumulates intracellularly in the Golgi but not in aggregates containing p62, suggesting that Atg7 may be involved in the transport of Aβ for degradation [49]. Moreover, in the 5xFAD transgenic mice model, Atg7 deletion impaired the microglial response to amyloid plaques [132].

### Glycogen synthase kinase 3β (GSK3β)

GSK3 is a serine threonine kinase that is involved in several signaling pathways. It has two isoforms: GSK3α and GSK3β. GSK3β regulates autophagy flux and was shown to be active in AD brains [133]. GSK3β dysregulation affected Aβ and Tau accumulation and clearance [133]. Hyperactivation of GSK3β was demonstrated in 5xFAD transgenic mice which express a mutant form of human APP, and inhibition of GSK3β increased lysosomal acidity, reduced Aβ deposits and ameliorated cognitive deficits [134]. Parr et al. reported that in N_2_a cells stably expressing APP, inhibition of GSK3β increased the numbers of lysosomes, which led to APP degradation and Aβ reduction [135]. Moreover, Aβ accumulation in AD brains activated GSK3β which phosphorylates Tau proteins and destabilized microtubules, resulting in axonal damage and neuronal death [136].

## The effect of sex on autophagy and Alzheimer’s

Two-thirds of AD patients are women, and have a greater lifetime risk of developing AD compared to men (1 in 10) [137]. As summarized in a recent review [138] and in Table 2, several studies suggest a faster progression or more severe pathology in women. Mechanistic studies show sex-dependent differences in neuronal, glial, metabolic, and immunological responses. Estrogen (especially 17β-estradiol) has been shown to have a neuroprotective effects and promotes autophagy via AMPK activation, independent of mTOR in tauopathy models [139]. Loss of estrogen is associated with reduced autophagic flux, insulin resistance, poorer tau and Aβ clearance [140]. The decline of estrogen during the menopausal transition disrupts mitochondrial protection and protein clearance via estrogen receptors (ERα, ERβ, GPER) [141–144]. Unlike men, women undergo menopausal transition with fluctuating estrogen levels during perimenopause, beginning dysfunction in metabolic, inflammatory, and sensory-processing pathways [142]. Increasing evidence suggests that ovarian hormone loss during this stage contributes to female vulnerability to AD. Progesterone receptors (PR-A, PR-B, mPRs, and PGRMC1/2), which are also expressed in the brain, contribute to sex-dependent regulation of autophagy. After menopause, women exhibit up to tenfold higher levels of Luteinizing Hormone (LH) than men, and together with Follicle Stimulating Hormone (FSH), these gonadotropins exacerbate AD pathology, and FSH is specifically linked to cognitive impairment [145, 146]. In cell and animal models, estrogen reduces amyloid-β by stimulating amyloid precursor protein (APP)-containing vesicles from the Golgi, enhancing APP delivery to the cell surface [147]. Interestingly, women display a greater tau burden than men, and neurofibrillary tangles increase AD risk up to 20-fold in women, compared to approximately threefold in men [148]. Sex-based differences are suggested to mediate the effects of AD risk factors. Incidence of AD in diabetic women is higher than in men [149]. Another risk factor for AD, the lipid carrier APOE4 allele, is associated with a higher AD risk in females compared to males [150]. Women carrying a single copy of the APOE4 allele show earlier onset, reduced hippocampal volume, and worse cognition, while men require two copies to exhibit similar effects [151] (reviewed in [140]). Likewise, tau pathology shows pronounced sex differences. Development of neurofibrillary lesions increases the likelihood of displaying cognitive AD symptoms and this effect is more pronounced in women [148]. It was also shown that several autophagy/lysosomal regulating genes are X-linked (e.g. LAMP2, ATP6AP2, members of the Rab family) and may escape X-inactivation, resulting in dosage differences between males and females [138]. At the chromosomal level, X-linked escapee genes such as *USP11* increase tau acetylation and impair degradation, whereas *KDM6A* confers neuroprotection [152, 153]. Sex chromosomes may influence baseline levels of autophagy gene expression; e.g., PTEN is higher in female mouse cortex, Klotho is higher in male cortex [138]. Genes such as *PDK4* and *HK2*, which regulate metabolism, are also under sex-chromosome influence, affecting mitochondrial function and aging trajectories [154]. As nicely summarized by Lopez-Lee et al., sex differences result from the combined contribution of gonadal hormones and sex chromosomes to neuroinflammation, epigenetics, metabolism, autophagy, and other molecular mechanisms, including the microbiome [138]. In Table 1, we summarize the effects of sex and hormones on autophagy, microglial activity, inflammation, metabolism and mitochondrial functions. Neuroinflammation represents an additional major axis of sex differences: microglial number, phagocytic capacity, and gene expression variation between the sexes, with male microglia showing higher baseline MHC-I expression, whereas female microglia display differential responses under pathological conditions. Metabolic pathways mirror these differences; estrogen promotes mitochondrial biogenesis and glycolytic flexibility, while its loss during menopause predisposes women to metabolic dysregulation, including age-associated insulin resistance, which further exacerbates neuronal vulnerability and accelerates AD-related pathology [155–158]. At the cellular level, it has been shown that in female 3xTg-AD mice, autophagosome degradation was impaired and resulted in LC3B-II and SQSTM1 accumulation in the cortex, while in males the levels of LC3B-II were reduced without major degradation deficits. Likewise, mitophagy is sex-biased with upregulated *BNIP3L* and *BCL2L13* expression in females yet still exhibited mitochondrial dysfunction and reduced mitochondrial number. These molecular patterns translate into distinct behavioral phenotypes, with females demonstrating impaired non-spatial memory which may be associated with mitochondrial turnover failure, while in males, preserved autophagic flux was associated with recognition memory [158]. Sex differences in mitophagy/mitochondrial quality control have also been described. Female mice showed accumulation of autophagosomes, indicating impaired degradation, while male mice showed low levels of autophagosomes and elevated mitophagy. These differences correlated with impaired mitochondrial function and cognitive deficits in females [158].

**Table 2:** Sex differences in autophagy pathways and their contribution to AD

Category and key elements	Sex specificity and differential impact	References
Sex hormones		
Estrogen	Females: loss of estrogen at menopause (systemic and mitochondrial). Supports mitochondrial function and reduces Tau accumulation. Its loss increases brain vulnerability	[138, 198–200]
Testosterone	Males: Declin gradually with age. The testosterone decrease is less abrupt than the estrogen thus it affects resilience	[138, 144, 201, 202]
Progesterone	Females and males: Widespread CNS expression, influences autophagy via multiple pathways. Lower levels of progesterone are associated with increased neurodegeneration and AD risk	[202]
FSH (Follicle stimulating hormone)	Females: post-menopause increase. FSH facilitate AD pathology through autophagy	[146, 203]

Sex chromosomes		
USP11 (X-linked, tau acetylation)	Females: X-linked, tau pathology. Increases tau acetylation, impairs clearance. Stronger effect in females	[152]
KDM6A (X-linked, neuroprotection)	Females: X-linked gene. Have a protective effect, it is associated with reduced mortality and cognitive protection	[153]
IL2RG, RAB9A and EMD. (X-linked, neurodegeneration)	Females: X-linked gene. cognitive decline and Tau pathology	[138]
Mosaic Y loss (Reduction in the levels of TMSB4X/Y, IL9R, CSF2RA)	Males: (Y loss in immune cells is linked to immune dysfunction and higher AD risk	[204]

Autophagy/ mitophagy markers		
LC3B-II (Atg8)	Females: Higher accumulation in the cortex, impaired protein degradation Males: reduced levels in the cortex and hippocampus	[158]
SQSTM1/p62	Females: Elevated levels in the cortex, impaired degradation	[158]
Beclin 1	Females: decreased expression and increases protein accumulation	[121, 205]
BNIP3	Males: Higher dimer levels compared to females, linked to resilience in males and impaired mitophagy in females	[158]
BNIP3L (NIX)	Females: Elevated levels of the monomers, increased mitochondrial damage and enhanced mitophagy	[158]
BCL2L13	Females: Elevated levels, enhanced mitophagy and memory decline	[158]

Immune/inflammation		
Microglial activity	Males: more amoeboid morphology and reduced plaque load Females: increased glycolysis and reduced ability to clear amyloid plaques	[206–208]
TREM2/TYROBP, CX3CR1 pathways	Females: exhibit stronger microglial activation and neuroinflammation	[209, 210]

Metabolism		
Mitochondrial estrogen receptors	Females: lower levels in female AD patients are associated with mitochondrial dysfunction	[211–213]
Brain glucose metabolism (insulin resistance)	Females: metabolic dysregulations and greater microglial activation that could contribute to accelerated cognitive decline	[214]

Genetic risk factor		
APOE4	Females: Increases AD risk, and pathological and cognitive effects	[215]

## The link between autophagy/mitophagy, microglia, biomarkers and lifestyle

As described above mitophagy is the specific form of autophagy, destined to clear damaged mitochondria (via PINK1/Parkin, BNIP3L, p62, LC3, etc.) [159]. Mitochondrial dysfunction is characterized among others by ROS elevation, mtDNA damage and loss of mitochondrial membrane potential, and is a core feature of AD pathogenesis [160]. In AD, there is evidence of upregulation of early mitophagy markers (PINK1, BNIP3L) and decreases in master regulators of lysosomal and mitochondrial biogenesis, such as TFEB in later stages, suggesting an impairment in the latter stages of mitophagy/autophagy-lysosomal clearance [160]. In astrocytes, it has been demonstrated that expression of *APOE4* impairs autophagy, mitophagy and mitochondrial functions [13, 89, 91]. Autophagy in microglia regulates phagocytic removal of debris (Aβ, apoptotic cells) and modulates inflammatory responses (e.g., via the NLRP3 inflammasome). Dysregulated microglial autophagy can lead to excessive neuroinflammation [161, 162]. Also, autophagy is regulated by several AD-related microglial receptors, (TLR4, TREM2, P2X7R, and CD36), mainly via regulation of mTOR, which affects autophagy induction [161].

Studies have shown that biomarkers of autophagy/mitophagy (ATG5 and Parkin) are reduced in the sera of patients with AD and Mild Cognitive Impairment (MCI) compared to controls [163]. Also, a study was shown correlation between changes in mitophagy biomarkers in CSF (PINK1, a mitochondrial kinase crucial for mitophagy) and in serum (BNIP3L, a mitophagy receptor; TFEB, a transcription factor critical for lysosomal degradation) and with markers of neurodegeneration (NfL), synaptic dysfunction (neurogranin), and cognitive decline [160]. Growing indications exist to suggest that diet and nutrients can affect autophagy. Autophagy is important for normal cellular function, and defective autophagy can result in various metabolic abnormalities and thus affect AD. Caloric restriction, fasting, or specific nutrient limitation can upregulate autophagy and mitigate AD-like pathology in animal models. It was demonstrated that compounds that enhance mitophagy/autophagy pathways (resveratrol, spermidine, urolithin A, NAD^+^) can suppress APP/Aβ-induced and mutant Tau-induced mitochondrial and synaptic dysfunctions in mouse and cell line models of AD [164]. Furthermore, lifestyle and exercise are known to improve mitochondria biogenesis and are associated autophagy activation [165, 166]. Disrupted sleep/circadian rythemus can aggravate many disease development including AD [167, 168]. Sleep disorders can also impair autophagy, enhance cellular waste accumulation, increase oxidative stress and impair autophagy. Conversely, adequate sleep and aligned circadian rhythms may facilitate effective autophagic flux [168, 169].

## Treatments that target autophagy in AD

**Fig. 3:**
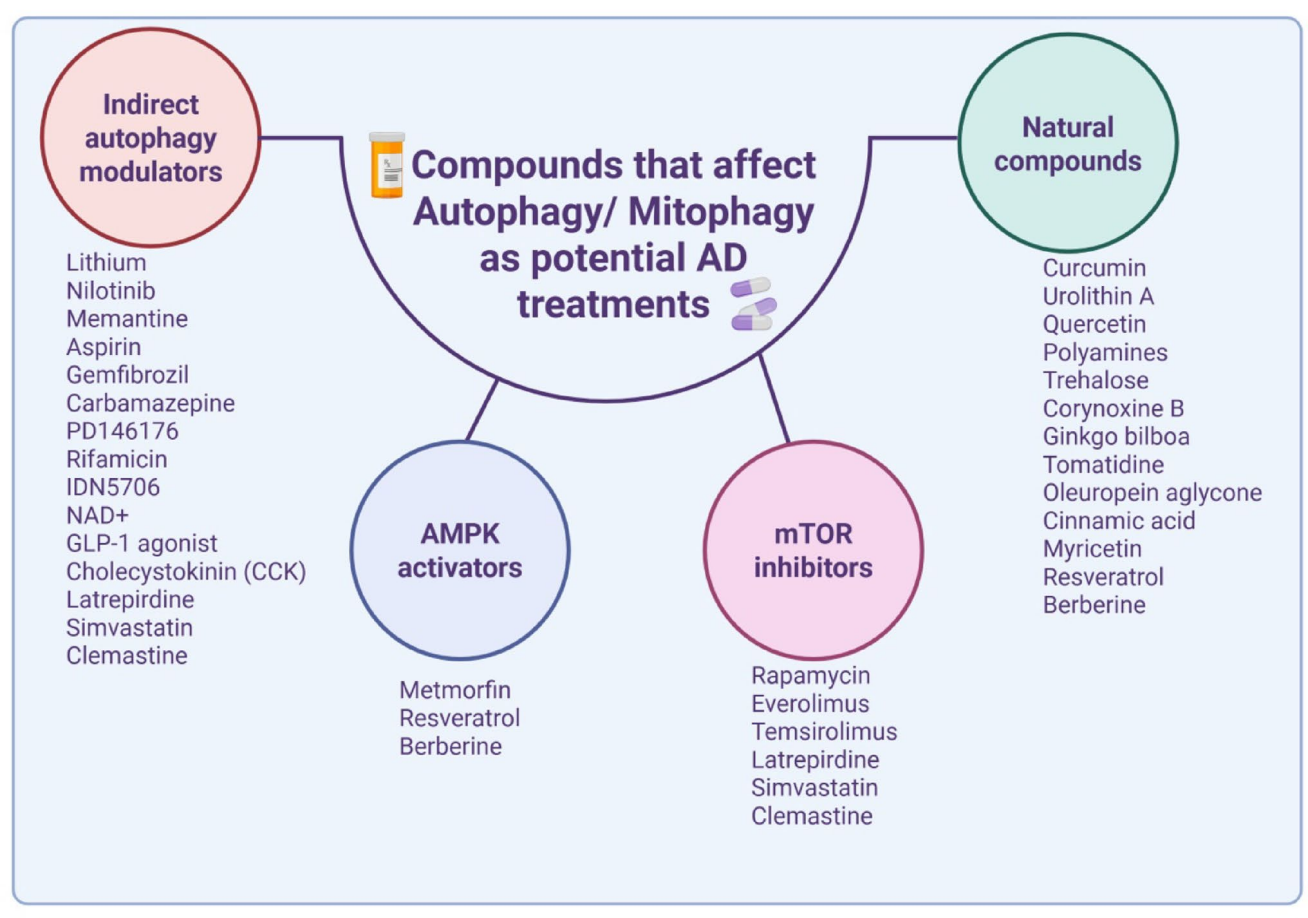
The scheme describes possible therapeutic approaches for AD that target autophagy related pathways. Specifically, it presents various strategies aimed at enhancing autophagy in order to support neuronal survival. These approaches include indirect autophagy modulators, AMPK activators, mTOR inhibitors and natural compounds

Several autophagy-enhancing agents have been suggested as therapeutic approaches for AD (Fig. 3). These include drugs that affect the mTOR signaling pathway, such as rapamycin, an mTOR inhibitor that induces autophagic flux and promotes neuroprotection by clearing Aβ and pTau oligomers [170]. Moreover, other mTOR inhibitors (Everolimus, Temsirolimus, Latrepirdine, Simvastatin and Clemastine) were shown to reduce Aβ load and increase autophagy [171–174]. Other drugs that affect autophagy via AMPK-ULK1 activation include Resveratrol, Berberine and Metformin which also regulate the activity of lysosomal protein cathepsin D and facilitate aggregate clearance [175–177]. There are several indirect autophagy modulators that affect autophagy flux and neuroprotection. Bonavita et al. focused on small heat shock proteins (sHSPs) as a potential therapeutic targets for aggregation pathologies, since they affect autophagy as well as the secretion and spreading of protein aggregates by extracellular vesicles [178]. Lithium also engages autophagy for neuroprotection [179]. In addition, the long non-coding RNA LINC00672 has been identified as a regulator of autophagy in neurons through upregulation of glycoprotein non-metastatic melanoma protein B (GPNMB). This LINC00672–GPNMB axis promotes autophagosome formation and facilitates the clearance of Aβ and pTau, offering a novel molecular target for restoring neuronal homeostasis in AD [180]. Another approach involves targeting lysosomal function. Nilotinib, an approved FDA drug for chronic myeloid leukemia, inhibits ABL1, a tyrosine kinase that impairs lysosomal function, thereby enhancing autophagy. This drug was shown to improve Aβ clearance and memory deficits in Tg2576 mice [175]. Gemfibrozil, a PPARα agonist promotes autophagy and the removal of Aβ plaques from the hippocampus and cortex of AD mouse models [177]. Also, Acetylsalicylic acid (aspirin) was shown to induces lysosomal biogenesis and attenuates AD pathology [181]. It was also shown that Memantine, an N-methyl-D-aspartate receptor inhibitor, has an anti-autophagic and anti-apoptotic effects supporting its potential as a new potential drug [182]. Additional compound, Quercetin, targets SIRT1 and Keap1/Nrf2/HO-1 pathways. Studies indicate that quercetin enhances autophagosome-lysosome fusion, promotes Aβ clearance and protects SH-SY5Y cells from Aβ-induced damage [183]. Another approach to affect autophagy as AD treatment includes exosomes, due to their ability to cross the blood–brain barrier [184]. Lyaswamy et al. engineered Fe65-overexpressing exosomes (Fe65-EXO) from hippocampal neurons to deliver Corynoxine-B (Cory-B), an autophagy inducer, specifically to APP overexpressing neurons of 5xFAD mice. This treatment improved cognitive decline, alleviated Aβ plaques and reduced pTau accumulation [184]. Other autophagy/mitophagy inducers that may affect amyloid pathology were described including: the natural disaccharide-trehalose, cinnamic acid derivates, tomatidine, oleuropein aglycone, curcumin and its analogs, ginkgo biloba extract, arctigenin extracted from Arctium lappa seeds [171, 172, 185–187]. PD146176 is a 12/15-lipoxygenase (12/15-LO) inhibitor that has been shown to reverse cognitive impairment and pathology in mouse models of AD by indirectly stimulating autophagy [188]. Further studies are required to explore the therapeutic potential of autophagy regulation for AD treatment.

## Conclusion and perspectives

AD neuropathology is characterized by the accumulation of Aβ plaques and neurofibrillary tangles (NFTs) [33, 54]. A great effort has been made to develop treatments that decrease tauopathies and amyloidosis and reduce the AD symptoms. In the present review we describe autophagy, a degradation pathway through the lysosome that maintains cell homeostasis [7, 16]. A growing body of evidence indicates that dysfunctional autophagy correlates with increased AD pathologies. Moreover, it is likely that autophagy can be a major contributor to AD development. Several AD related risk factor proteins and peptides, including PSEN1, TREM2, Aβ and APOE have been demonstrated to affect autophagic flux. Also, several autophagy regulating genes including *Atg7*, Beclin1, LC3, p62, etc. were demonstrated to be altered in AD animal models and AD patient samples. Thus, enhancing autophagy to eliminate misfolded proteins proposed as a potential AD therapy. Indeed, a variety of autophagy enhancers have been shown to slow AD progression and to improve cognition, at least in AD animal models. However, the beneficial effects of autophagy stimulators in AD patients have not been observed or are limited. We describe several autophagy related proteins that were implicated in AD pathology. In addition, we describe studies showing that Aβ plaques and neurofibrillary tangles (NFTs) are a consequence of impaired autophagy [78]. Taken together, this review implicates the role of autophagy in AD pathology and suggests that further research into autophagy may lead to more therapeutic approaches.

## Data Availability

No datasets were generated or analysed during the current study.

## References

[1] Aathira NS, Kaur A, Kumar A, Dar GM, Nimisha, Sharma AK, et al. The genetic risk factors, molecular pathways, microRNAs, and the gut microbiome in Alzheimer’s disease. Neuroscience. 2025;577:217–27. 10.1016/j.neuroscience.2025.05.021. 40374065 10.1016/j.neuroscience.2025.05.021

[2] Biamonti G, Amato A, Belloni E, Di Matteo A, Infantino L, Pradella D, et al. Alternative splicing in Alzheimer’s disease. Aging Clin Exp Res. 2021;33:747–58. 10.1007/s40520-019-01360-x. 31583531 10.1007/s40520-019-01360-x

[3] Zhao N, Liu C-C, Qiao W, Bu G. Apolipoprotein E, receptors, and modulation of Alzheimer’s disease. Biol Psychiat. 2018;83:347–57. 10.1016/j.biopsych.2017.03.003. 28434655 10.1016/j.biopsych.2017.03.003 PMC5599322

[4] Ballard C, Gauthier S, Corbett A, Brayne C, Aarsland D, Jones E. Alzheimer’s disease. Lancet. 2011;377:1019–31. 10.1016/S0140-6736(10)61349-9. 21371747 10.1016/S0140-6736(10)61349-9

[5] Fedeli C, Filadi R, Rossi A, Mammucari C, Pizzo P. PSEN2 (presenilin 2) mutants linked to familial Alzheimer disease impair autophagy by altering Ca^2+^ homeostasis. Autophagy. 2019;15:2044–62. 10.1080/15548627.2019.1596489. 30892128 10.1080/15548627.2019.1596489 PMC6844518

[6] Dai M-H, Zheng H, Zeng L-D, Zhang Y. The genes associated with early-onset Alzheimer’s disease. Oncotarget. 2018;9:15132–43. 10.18632/oncotarget.23738. 29599933 10.18632/oncotarget.23738 PMC5871104

[7] Levine B, Kroemer G. Biological functions of autophagy genes: a disease perspective. Cell. 2019;176:11–42. 10.1016/j.cell.2018.09.048. 30633901 10.1016/j.cell.2018.09.048 PMC6347410

[8] Zhang Z, Yang X, Song Y-Q, Tu J. Autophagy in Alzheimer’s disease pathogenesis: therapeutic potential and future perspectives. Ageing Res Rev. 2021;72:101464. 10.1016/j.arr.2021.101464. 34551326 10.1016/j.arr.2021.101464

[9] Komatsu M, Waguri S, Chiba T, Murata S, Iwata J, Tanida I, et al. Loss of autophagy in the central nervous system causes neurodegeneration in mice. Nature. 2006;441:880–4. 10.1038/nature04723. 16625205 10.1038/nature04723

[10] Hara T, Nakamura K, Matsui M, Yamamoto A, Nakahara Y, Suzuki-Migishima R, et al. Suppression of basal autophagy in neural cells causes neurodegenerative disease in mice. Nature. 2006;441:885–9. 10.1038/nature04724. 16625204 10.1038/nature04724

[11] Nixon RA, Wegiel J, Kumar A, Yu WH, Peterhoff C, Cataldo A, et al. Extensive involvement of autophagy in Alzheimer disease: an immuno-electron microscopy study. J Neuropathol Exp Neurol. 2005;64:113–22. 15751225 10.1093/jnen/64.2.113

[12] Kim S, Chun H, Kim Y, Kim Y, Park U, Chu J, et al. Astrocytic autophagy plasticity modulates Aβ clearance and cognitive function in Alzheimer’s disease. Mol Neurodegener. 2024;19:55. 10.1186/s13024-024-00740-w. 39044253 10.1186/s13024-024-00740-w PMC11267931

[13] Simonovitch S, Schmukler E, Bespalko A, Iram T, Frenkel D, Holtzman DM, et al. Impaired autophagy in APOE4 astrocytes. JAD. 2016;51:915–27. 10.3233/JAD-151101. 26923027 10.3233/JAD-151101

[14] Chong C-M, Ke M, Tan Y, Huang Z, Zhang K, Ai N, et al. Presenilin 1 deficiency suppresses autophagy in human neural stem cells through reducing γ-secretase-independent ERK/CREB signaling. Cell Death Dis. 2018;9:879. 10.1038/s41419-018-0945-7. 30158533 10.1038/s41419-018-0945-7 PMC6115391

[15] Krishnamurthy HK, Jayaraman V, Krishna K, Wang T, Bei K, Changalath C, et al. An overview of the genes and biomarkers in Alzheimer’s disease. Ageing Res Rev. 2025;104:102599. 10.1016/j.arr.2024.102599. 39612989 10.1016/j.arr.2024.102599

[16] Glick D, Barth S, Macleod KF. Autophagy: cellular and molecular mechanisms. J Pathol. 2010;221:3–12. 10.1002/path.2697. 20225336 10.1002/path.2697 PMC2990190

[17] Nixon RA, Rubinsztein DC. Mechanisms of autophagy–lysosome dysfunction in neurodegenerative diseases. Nat Rev Mol Cell Biol. 2024;25:926–46. 10.1038/s41580-024-00757-5. 39107446 10.1038/s41580-024-00757-5 PMC12239022

[18] Korolchuk VI, Sarkar S, Fanto M. Autophagy in neurodegenerative diseases. J Mol Biol. 2020;432:2445–8. 10.1016/j.jmb.2020.03.005. 32169483 10.1016/j.jmb.2020.03.005

[19] Fleming A, Rubinsztein DC. Autophagy in neuronal development and plasticity. Trends Neurosci. 2020;43:767–79. 10.1016/j.tins.2020.07.003. 32800535 10.1016/j.tins.2020.07.003

[20] Nixon RA. Autophagy–lysosomal-associated neuronal death in neurodegenerative disease. Acta Neuropathol. 2024;148:42. 10.1007/s00401-024-02799-7. 39259382 10.1007/s00401-024-02799-7 PMC11418399

[21] Aman Y, Schmauck-Medina T, Hansen M, Morimoto RI, Simon AK, Bjedov I, et al. Autophagy in healthy aging and disease. Nat Aging. 2021;1:634–50. 10.1038/s43587-021-00098-4. 34901876 10.1038/s43587-021-00098-4 PMC8659158

[22] Yim WW-Y, Mizushima N. Lysosome biology in autophagy. Cell Discov. 2020;6:6. 10.1038/s41421-020-0141-7. 32047650 10.1038/s41421-020-0141-7 PMC7010707

[23] Karmacharya U, Jung J-W. Small molecule inhibitors for Unc-51-like autophagy-activating kinase targeting autophagy in cancer. IJMS. 2023;24:953. 10.3390/ijms24020953. 36674464 10.3390/ijms24020953 PMC9866249

[24] Russell RC, Tian Y, Yuan H, Park HW, Chang Y-Y, Kim J, et al. ULK1 induces autophagy by phosphorylating Beclin-1 and activating VPS34 lipid kinase. Nat Cell Biol. 2013;15:741–50. 10.1038/ncb2757. 23685627 10.1038/ncb2757 PMC3885611

[25] Ondaro J, Hernandez-Eguiazu H, Garciandia-Arcelus M, Loera-Valencia R, Rodriguez-Gómez L, Jiménez-Zúñiga A, et al. Defects of nutrient signaling and autophagy in neurodegeneration. Front Cell Dev Biol. 2022;10:836196. 10.3389/fcell.2022.836196. 35419363 10.3389/fcell.2022.836196 PMC8996160

[26] Walczak M, Martens S. Dissecting the role of the Atg12–Atg5-Atg16 complex during autophagosome formation. Autophagy. 2013;9:424–5. 10.4161/auto.22931. 23321721 10.4161/auto.22931 PMC3590266

[27] Lee Y-K, Lee J-A. Role of the mammalian ATG8/LC3 family in autophagy: differential and compensatory roles in the spatiotemporal regulation of autophagy. BMB Rep. 2016;49:424–30. 10.5483/BMBRep.2016.49.8.081. 27418283 10.5483/BMBRep.2016.49.8.081 PMC5070729

[28] Palmer JE, Wilson N, Son SM, Obrocki P, Wrobel L, Rob M, et al. Autophagy, aging, and age-related neurodegeneration. Neuron. 2025;113:29–48. 10.1016/j.neuron.2024.09.015. 39406236 10.1016/j.neuron.2024.09.015

[29] Ye J, Jiang Z, Chen X, Liu M, Li J, Liu N. The role of autophagy in pro-inflammatory responses of microglia activation via mitochondrial reactive oxygen species *in vitro*. J Neurochem. 2017;142:215–30. 10.1111/jnc.14042. 28407242 10.1111/jnc.14042

[30] Cho M-H, Cho K, Kang H-J, Jeon E-Y, Kim H-S, Kwon H-J, et al. Autophagy in microglia degrades extracellular β-amyloid fibrils and regulates the NLRP3 inflammasome. Autophagy. 2014;10:1761–75. 10.4161/auto.29647. 25126727 10.4161/auto.29647 PMC4198361

[31] Houtman J, Freitag K, Gimber N, Schmoranzer J, Heppner FL, Jendrach M. Beclin1-driven autophagy modulates the inflammatory response of microglia via NLRP 3. EMBO J. 2019;38:e99430. 10.15252/embj.201899430. 30617086 10.15252/embj.201899430 PMC6376276

[32] Glenner GG, Wong CW. Alzheimer’s disease: initial report of the purification and characterization of a novel cerebrovascular amyloid protein. Biochem Biophys Res Commun. 1984;120:885–90. 10.1016/S0006-291X(84)80190-4. 6375662 10.1016/s0006-291x(84)80190-4

[33] Masters CL, Simms G, Weinman NA, Multhaup G, McDonald BL, Beyreuther K. Amyloid plaque core protein in Alzheimer disease and down syndrome. Proc Natl Acad Sci USA. 1985;82:4245–9. 10.1073/pnas.82.12.4245. 3159021 10.1073/pnas.82.12.4245 PMC397973

[34] Murphy MP, LeVine H. Alzheimer’s disease and the amyloid-β peptide. J Alzheimer’s Dis. 2010;19:311–23. 10.3233/JAD-2010-1221. 20061647 10.3233/JAD-2010-1221 PMC2813509

[35] Orobets KS, Karamyshev AL. Amyloid precursor protein and Alzheimer’s disease. IJMS. 2023;24:14794. 10.3390/ijms241914794. 37834241 10.3390/ijms241914794 PMC10573485

[36] Schmitz C, Rutten BPF, Pielen A, Schäfer S, Wirths O, Tremp G, et al. Hippocampal neuron loss exceeds amyloid plaque load in a transgenic mouse model of Alzheimer’s disease. Am J Pathol. 2004;164:1495–502. 10.1016/S0002-9440(10)63235-X. 15039236 10.1016/S0002-9440(10)63235-X PMC1615337

[37] Nilsson P, Loganathan K, Sekiguchi M, Matsuba Y, Hui K, Tsubuki S, et al. Aβ secretion and plaque formation depend on autophagy. Cell Rep. 2013;5:61–9. 10.1016/j.celrep.2013.08.042. 24095740 10.1016/j.celrep.2013.08.042

[38] Sonoda R, Kuramoto E, Minami S, Matsumoto SE, Ohyagi Y, Saito T, et al. Reduced autophagy in aged trigeminal neurons causes amyloid β diffusion. J Dent Res. 2023;102:938–46. 10.1177/00220345231156095. 36919893 10.1177/00220345231156095

[39] Suelves N, Saleki S, Ibrahim T, Palomares D, Moonen S, Koper MJ, et al. Senescence-related impairment of autophagy induces toxic intraneuronal amyloid-β accumulation in a mouse model of amyloid pathology. Acta Neuropathol Commun. 2023;11:82. 10.1186/s40478-023-01578-x. 37198698 10.1186/s40478-023-01578-x PMC10189946

[40] Xin S-H, Tan L, Cao X, Yu J-T, Tan L. Clearance of amyloid beta and tau in Alzheimer’s disease: from mechanisms to therapy. Neurotox Res. 2018;34:733–48. 10.1007/s12640-018-9895-1. 29626319 10.1007/s12640-018-9895-1

[41] Singh AK, Bissoyi A, Kashyap MP, Patra PK, Rizvi SI. Autophagy activation alleviates amyloid-β-induced oxidative stress, apoptosis and neurotoxicity in human neuroblastoma SH-SY5Y cells. Neurotox Res. 2017;32:351–61. 10.1007/s12640-017-9746-5. 28484969 10.1007/s12640-017-9746-5

[42] Spilman P, Podlutskaya N, Hart MJ, Debnath J, Gorostiza O, Bredesen D, et al. Inhibition of mTOR by rapamycin abolishes cognitive deficits and reduces amyloid-β levels in a mouse model of Alzheimer’s disease. PLoS ONE. 2010;5:e9979. 10.1371/journal.pone.0009979. 20376313 10.1371/journal.pone.0009979 PMC2848616

[43] Liang Y, Zhou T, Chen Y, Lin D, Jing X, Peng S, et al. Rifampicin inhibits rotenone-induced microglial inflammation via enhancement of autophagy. Neurotoxicology. 2017;63:137–45. 10.1016/j.neuro.2017.09.015. 28986232 10.1016/j.neuro.2017.09.015

[44] Zhu L, Yuan Q, Zeng Z, Zhou R, Luo R, Zhang J, et al. Rifampicin suppresses amyloid-β accumulation through enhancing autophagy in the Hippocampus of a lipopolysaccharide-induced mouse model of cognitive decline. J Alzheimers Dis. 2021;79:1171–84. 10.3233/JAD-200690. 33386800 10.3233/JAD-200690

[45] Cavieres VA, González A, Muñoz VC, Yefi CP, Bustamante HA, Barraza RR, et al. Tetrahydrohyperforin inhibits the proteolytic processing of amyloid precursor protein and enhances its degradation by Atg5-dependent autophagy. PLoS ONE. 2015;10:e0136313. 10.1371/journal.pone.0136313. 26308941 10.1371/journal.pone.0136313 PMC4550396

[46] García-Juan M, Ordóñez-Gutiérrez L, Wandosell F. Clearance of β-amyloid mediated by autophagy is enhanced by MTORC1 inhibition but not AMPK activation in APP/PSEN1 astrocytes. Glia. 2024;72:588–606. 10.1002/glia.24492. 38009275 10.1002/glia.24492

[47] Benito-Cuesta I, Ordóñez-Gutiérrez L, Wandosell F. AMPK activation does not enhance autophagy in neurons in contrast to MTORC1 inhibition: different impact on β-amyloid clearance. Autophagy. 2021;17:656–71. 10.1080/15548627.2020.1728095. 32075509 10.1080/15548627.2020.1728095 PMC8032230

[48] Chen X, Muñoz-Arellano AJ, Petranovic D. UBB+1 reduces amyloid-β cytotoxicity by activation of autophagy in yeast. Aging. 2021;13:23953–80. 10.18632/aging.203681. 34751669 10.18632/aging.203681 PMC8610117

[49] Nilsson P, Sekiguchi M, Akagi T, Izumi S, Komori T, Hui K, et al. Autophagy-related protein 7 deficiency in amyloid β (Aβ) precursor protein transgenic mice decreases Aβ in the multivesicular bodies and induces Aβ accumulation in the Golgi. Am J Pathol. 2015;185:305–13. 10.1016/j.ajpath.2014.10.011. 25433221 10.1016/j.ajpath.2014.10.011

[50] Luo R, Su L-Y, Li G, Yang J, Liu Q, Yang L-X, et al. Activation of *PPARA*-mediated autophagy reduces Alzheimer disease-like pathology and cognitive decline in a murine model. Autophagy. 2020;16:52–69. 10.1080/15548627.2019.1596488. 30898012 10.1080/15548627.2019.1596488 PMC6984507

[51] De La Cueva M, Antequera D, Ordoñez-Gutierrez L, Wandosell F, Camins A, Carro E, et al. Amyloid-β impairs mitochondrial dynamics and autophagy in Alzheimer’s disease experimental models. Sci Rep. 2022;12:10092. 10.1038/s41598-022-13683-3. 35710783 10.1038/s41598-022-13683-3 PMC9203760

[52] Luo R, Kang Y, Ma H, Zhang Z, Hölscher C, Hao L, et al. A novel dual Cck/ Glp-1 receptor agonist ameliorates cognitive impairment in 5×Fad mice by modulating mitophagy via the Pink1/Parkin pathway. Int Immunopharmacol. 2025;154:114612. 40184808 10.1016/j.intimp.2025.114612

[53] Dou J, Su P, Xu C, Wen Z, Mao Z, Li W. Targeting Hsc70-based autophagy to eliminate amyloid β oligomers. Biochem Biophys Res Commun. 2020;524:923–8. 10.1016/j.bbrc.2020.02.016. 32057360 10.1016/j.bbrc.2020.02.016 PMC7085976

[54] Hamano T, Enomoto S, Shirafuji N, Ikawa M, Yamamura O, Yen S-H, et al. Autophagy and tau protein. Int J Mol Sci. 2021;22:7475. 10.3390/ijms22147475. 34299093 10.3390/ijms22147475 PMC8303176

[55] Grundke-Iqbal I, Wisniewski HM, Bindert LI. Abnormal phosphorylation of the microtubule-associated protein X (tau) in Alzheimer cytoskeletal pathology. Med Sci. 1986;83:4913–7. 10.1073/pnas.83.13.4913 PMC323854 3088567

[56] Medeiros R, Baglietto-Vargas D, LaFerla FM. The role of tau in Alzheimer’s disease and related disorders. CNS Neurosci Ther. 2011;17:514–24. 10.1111/j.1755-5949.2010.00177.x. 20553310 10.1111/j.1755-5949.2010.00177.x PMC4072215

[57] Goedert M. TauproteinandtheneurolbrillarypathologyofAIzheimer’s disease.

[58] Li H-L, Wang H-H, Liu S-J, Deng Y-Q, Zhang Y-J, Tian Q, et al. Phosphorylation of tau antagonizes apoptosis by stabilizing β-catenin, a mechanism involved in Alzheimer’s neurodegeneration. Proc Natl Acad Sci USA. 2007;104:3591–6. 10.1073/pnas.0609303104. 17360687 10.1073/pnas.0609303104 PMC1805527

[59] Caballero B, Bourdenx M, Luengo E, Diaz A, Sohn PD, Chen X, et al. Acetylated tau inhibits chaperone-mediated autophagy and promotes tau pathology propagation in mice. Nat Commun. 2021;12:1–18. 10.1038/s41467-021-22501-9. 33854069 10.1038/s41467-021-22501-9 PMC8047017

[60] Bramblett GT, Coedert M, Jakes R, Merrick SE, Trojanowski JQ. Abnormal Tau phosphorylation at Ser3g6 in Alzheimer’s disease recapitulates development and contributes to reduced microtubule binding. 10.1016/0896-6273(93)90057-x 8318230

[61] Chatterjee S, Sealey M, Ruiz E, Pegasiou CM, Brookes K, Green S, et al. Age-related changes in tau and autophagy in human brain in the absence of neurodegeneration. PLoS ONE. 2023;18:e0262792. 10.1371/journal.pone.0262792. 36701399 10.1371/journal.pone.0262792 PMC9879510

[62] Zheng J, Wang Y, Liu Y, Han S, Zhang Y, Luo Y, et al. cPKCγ deficiency exacerbates autophagy impairment and hyperphosphorylated tau buildup through the AMPK/mTOR pathway in mice with type 1 diabetes mellitus. Neurosci Bull. 2022;38:1153–69. 10.1007/s12264-022-00863-4. 35596894 10.1007/s12264-022-00863-4 PMC9554100

[63] Zhang T, Tian Y, Zheng X, Li R, Hu L, Shui X, et al. Activation of transient receptor potential vanilloid 1 ameliorates tau accumulation-induced synaptic damage and cognitive dysfunction via autophagy enhancement. CNS Neurosci Ther. 2024;30:e14432. 10.1111/cns.14432. 37641913 10.1111/cns.14432 PMC10916438

[64] Gao J, Chen X, Ma T, He B, Li P, Zhao Y, et al. PEG-ceramide nanomicelles induce autophagy and degrade tau proteins in N2a cells. IJN. 2020;15:6779–89. 10.2147/IJN.S258311. 32982233 10.2147/IJN.S258311 PMC7494393

[65] Kim S, Lee D, Song JC, Cho S-J, Yun S-M, Koh YH, et al. NDP52 associates with phosphorylated tau in brains of an Alzheimer disease mouse model. Biochem Biophys Res Commun. 2014;454:196–201. 10.1016/j.bbrc.2014.10.066. 25450380 10.1016/j.bbrc.2014.10.066

[66] Mattioni A, Carsetti C, Bruqi K, Caputo V, Cianfanelli V, Bacalini MG, et al. A variant of the autophagic receptor NDP52 counteracts phospho-TAU accumulation and emerges as a protective factor for Alzheimer’s disease. Cell Death Dis. 2025;16:300. 10.1038/s41419-025-07611-2. 40234443 10.1038/s41419-025-07611-2 PMC12000434

[67] Xu Y, Liu Y, Chen X, Xu Q, Liu L, Liu H, et al. OPTN attenuates the neurotoxicity of abnormal Tau protein by restoring autophagy. Transl Psychiatry. 2022;12:230. 10.1038/s41398-022-02004-x. 35662233 10.1038/s41398-022-02004-x PMC9167278

[68] Ferrari V, Cristofani R, Tedesco B, Crippa V, Chierichetti M, Casarotto E, et al. Valosin containing protein (VCP): A multistep regulator of autophagy. IJMS. 2022;23:1939. 10.3390/ijms23041939. 35216053 10.3390/ijms23041939 PMC8878954

[69] Berger Z, Ravikumar B, Menzies FM, Oroz LG, Underwood BR, Pangalos MN, et al. Rapamycin alleviates toxicity of different aggregate-prone proteins. Hum Mol Genet. 2006;15:433–42. 10.1093/hmg/ddi458. 16368705 10.1093/hmg/ddi458

[70] Silva MC, Nandi GA, Tentarelli S, Gurrell IK, Jamier T, Lucente D, et al. Prolonged tau clearance and stress vulnerability rescue by pharmacological activation of autophagy in tauopathy neurons. Nat Commun. 2020;11:3258. 10.1038/s41467-020-16984-1. 32591533 10.1038/s41467-020-16984-1 PMC7320012

[71] Sweeney N, Kim TY, Morrison CT, Li L, Acosta D, Liang J, et al. Neuronal BAG3 attenuates tau hyperphosphorylation, synaptic dysfunction, and cognitive deficits induced by traumatic brain injury via the regulation of autophagy-lysosome pathway. Acta Neuropathol. 2024;148:52. 10.1007/s00401-024-02810-1. 39394356 10.1007/s00401-024-02810-1 PMC11469979

[72] Ji C, Tang M, Zeidler C, Höhfeld J, Johnson GV. BAG3 and SYNPO (synaptopodin) facilitate phospho-MAPT/Tau degradation via autophagy in neuronal processes. Autophagy. 2019;15:1199–213. 10.1080/15548627.2019.1580096. 30744518 10.1080/15548627.2019.1580096 PMC6613891

[73] Dai B, Zhong T, Chen Z, Chen W, Zhang N, Liu X, et al. Myricetin slows liquid–liquid phase separation of Tau and activates ATG5-dependent autophagy to suppress Tau toxicity. J Biol Chem. 2021;297:101222. 10.1016/j.jbc.2021.101222. 34560101 10.1016/j.jbc.2021.101222 PMC8551527

[74] Kang S, Son SM, Baik SH, Yang J, Mook-jung I. Autophagy-mediated secretory pathway is responsible for both normal and pathological Tau in neurons. J Alzheimer’s Dis. 2019;70:667–80. 10.3233/JAD-190180. 31256134 10.3233/JAD-190180

[75] Falcon B, Noad J, McMahon H, Randow F, Goedert M. Galectin-8–mediated selective autophagy protects against seeded tau aggregation. J Biol Chem. 2018;293:2438–51. 10.1074/jbc.M117.809293. 29282296 10.1074/jbc.M117.809293 PMC5818177

[76] Pollack SJ, Dakkak D, Guo T, Chennell G, Gomez-Suaga P, Noble W, et al. Truncated tau interferes with the autophagy and endolysosomal pathway and results in lipid accumulation. Cell Mol Life Sci. 2024;81:304. 10.1007/s00018-024-05337-6. 39009859 10.1007/s00018-024-05337-6 PMC11335226

[77] Ferrari L, Bauer B, Qiu Y, Schuschnig M, Klotz S, Anrather D, et al. Tau fibrils evade autophagy by excessive p62 coating and TAX1BP1 exclusion. Sci Adv. 2024;10:8449. 10.1126/sciadv.adm8449 PMC11168460 38865459

[78] Feng Q, Luo Y, Zhang X-N, Yang X-F, Hong X-Y, Sun D-S, et al. MAPT/Tau accumulation represses autophagy flux by disrupting IST1-regulated ESCRT-III complex formation: a vicious cycle in Alzheimer neurodegeneration. Autophagy. 2020;16:641–58. 10.1080/15548627.2019.1633862. 31223056 10.1080/15548627.2019.1633862 PMC7138218

[79] Muñoz SS, Garner B, Ooi L. Understanding the role of ApoE fragments in Alzheimer’s disease. Neurochem Res. 2019;44:1297–305. 10.1007/s11064-018-2629-1. 30225748 10.1007/s11064-018-2629-1

[80] Strittmatter WJ, Enghild J, Salvesen GS, Roses AD. Apolipoprotein E: high-avidity binding to, B-amyloid and increased frequency of type 4 allele in late-onset familial Alzheimer disease. Proc NatL Acad Sci USA. 1993;90:1977–81. 8446617 10.1073/pnas.90.5.1977 PMC46003

[81] Van Der Lee SJ, Wolters FJ, Ikram MK, Hofman A, Ikram MA, Amin N, et al. The effect of APOE and other common genetic variants on the onset of Alzheimer’s disease and dementia: a community-based cohort study. Lancet Neurol. 2018;17:434–44. 10.1016/S1474-4422(18)30053-X. 29555425 10.1016/S1474-4422(18)30053-X

[82] Christensen DZ, Schneider-Axmann T, Lucassen PJ, Bayer TA, Wirths O. Accumulation of intraneuronal Aβ correlates with ApoE4 genotype. Acta Neuropathol. 2010;119:555–66. 10.1007/s00401-010-0666-1. 20217101 10.1007/s00401-010-0666-1 PMC2849938

[83] Yamazaki Y, Zhao N, Caulfield TR, Liu C-C, Bu G. Apolipoprotein E and Alzheimer disease: pathobiology and targeting strategies. Nat Rev Neurol. 2019;15:501–18. 10.1038/s41582-019-0228-7. 31367008 10.1038/s41582-019-0228-7 PMC7055192

[84] Raulin A-C, Doss SV, Trottier ZA, Ikezu TC, Bu G, Liu C-C. ApoE in Alzheimer’s disease: pathophysiology and therapeutic strategies. Mol Neurodegener. 2022;17:72. 10.1186/s13024-022-00574-4. 36348357 10.1186/s13024-022-00574-4 PMC9644639

[85] Alzheimer’s Disease Neuroimaging Initiative, Shi Y, Yamada K, Liddelow SA, Smith ST, Zhao L, et al. ApoE4 markedly exacerbates tau-mediated neurodegeneration in a mouse model of tauopathy. Nature. 2017;549:523–7. 10.1038/nature24016. 28959956 10.1038/nature24016 PMC5641217

[86] Lv S, Zhang Y, Lin Y, Fang W, Wang Y, Li Z, et al. ApoE4 exacerbates the senescence of hippocampal neurons and spatial cognitive impairment by downregulating acetyl-CoA level. Aging Cell. 2023;22:e13932. 10.1111/acel.13932. 37594184 10.1111/acel.13932 PMC10497817

[87] Parcon PA, Balasubramaniam M, Ayyadevara S, Jones RA, Liu L, Shmookler Reis RJ, et al. Apolipoprotein E4 inhibits autophagy gene products through direct, specific binding to CLEAR motifs. Alzheimer’s Dementia. 2018;14:230–42. 10.1016/j.jalz.2017.07.754. 28945989 10.1016/j.jalz.2017.07.754 PMC6613789

[88] Bassal R, Rivkin-Natan M, Rabinovich A, Michaelson DM, Frenkel D, Pinkas-Kramarski R. APOE4 impairs autophagy and Aβ clearance by microglial cells. Inflamm Res. 2025;74:61. 10.1007/s00011-025-02016-5. 40164781 10.1007/s00011-025-02016-5 PMC11958439

[89] Simonovitch S, Schmukler E, Masliah E, Pinkas-Kramarski R, Michaelson DM. The effects of APOE4 on mitochondrial dynamics and proteins *in vivo*. JAD. 2019;70:861–75. 10.3233/JAD-190074. 31306119 10.3233/JAD-190074 PMC7478177

[90] Schmukler E, Michaelson DM, Pinkas-Kramarski R. The interplay between apolipoprotein E4 and the autophagic–endocytic–lysosomal axis. Mol Neurobiol. 2018;55:6863–80. 10.1007/s12035-018-0892-4. 29353455 10.1007/s12035-018-0892-4

[91] Schmukler E, Solomon S, Simonovitch S, Goldshmit Y, Wolfson E, Michaelson DM, et al. Altered mitochondrial dynamics and function in APOE4-expressing astrocytes. Cell Death Dis. 2020;11:578. 10.1038/s41419-020-02776-4. 32709881 10.1038/s41419-020-02776-4 PMC7382473

[92] Butt OH, Long JM, Henson RL, Herries E, Sutphen CL, Fagan AM, et al. Cognitively normal APOE ε4 carriers have specific elevation of CSF SNAP-25. Neurobiol Aging. 2021;102:64–72. 10.1016/j.neurobiolaging.2021.02.008. 33765432 10.1016/j.neurobiolaging.2021.02.008 PMC8793109

[93] Sohn H-Y, Kim S-I, Park J-Y, Park S-H, Koh YH, Kim J, et al. ApoE4 attenuates autophagy via FoxO3a repression in the brain. Sci Rep. 2021;11(1):17604. 10.1038/s41598-021-97117-6. 34475505 10.1038/s41598-021-97117-6 PMC8413297

[94] Chen Y, Song S, Parhizkar S, Lord J, Zhu Y, Strickland MR, et al. APOE3ch alters microglial response and suppresses Aβ-induced tau seeding and spread. Cell. 2024;187:428-445.e20. 10.1016/j.cell.2023.11.029. 38086389 10.1016/j.cell.2023.11.029 PMC10842861

[95] Fote GM, Steffan JS. APOE4 dysregulates autophagy in cultured cells. Autophagy Rep. 2022;1:29–33. 10.1080/27694127.2022.2040767. 38912292 10.1080/27694127.2022.2040767 PMC11192451

[96] Takasugi N, Tomita T, Hayashi I, Tsuruoka M, Niimura M, Takahashi Y, et al. The role of presenilin cofactors in the γ-secretase complex. Nature. 2003. 10.1038/nature01506. 12660785 10.1038/nature01506

[97] Wolfe MS, Xia W, Ostaszewski BL, Diehl TS, Kimberly WT, Selkoe DJ. Two transmembrane aspartates in presenilin-1 required for presenilin endoproteolysis and g-secretase activity. Nature. 1999;398:513–7. 10206644 10.1038/19077

[98] Bentahir M, Nyabi O, Verhamme J, Tolia A, Horré K, Wiltfang J, et al. Presenilin clinical mutations can affect γ-secretase activity by different mechanisms. J Neurochem. 2006;96:732–42. 10.1111/j.1471-4159.2005.03578.x. 16405513 10.1111/j.1471-4159.2005.03578.x

[99] Farfara D, Trudler D, Segev-Amzaleg N, Galron R, Stein R, Frenkel D. γ-secretase component presenilin is important for microglia β-amyloid clearance. Ann Neurol. 2011;69:170–80. 10.1002/ana.22191. 21280087 10.1002/ana.22191

[100] Jayadev S, Case A, Eastman AJ, Nguyen H, Pollak J, Wiley JC, et al. Presenilin 2 is the predominant γ-secretase in microglia and modulates cytokine release. PLoS ONE. 2010;5:e15743. 10.1371/journal.pone.0015743. 21206757 10.1371/journal.pone.0015743 PMC3012089

[101] Lauritzen I, Bini A, Bécot A, Gay A, Badot C, Pagnotta S, et al. Presenilins as hub proteins controlling the endocytic and autophagic pathways and small extracellular vesicle secretion. J Extracell Vesicle. 2025;14:e70019. 10.1002/jev2.70019. 10.1002/jev2.70019 PMC11735957 39815792

[102] Neely KM, Green KN, LaFerla FM. Presenilin Is necessary for efficient proteolysis through the autophagy-lysosome system in a γ-secretase-independent manner. J Neurosci. 2011;31:2781–91. 10.1523/JNEUROSCI.5156-10.2010. 21414900 10.1523/JNEUROSCI.5156-10.2010 PMC3064964

[103] Journal of Neurochemistry—2006—Bentahir—Presenilin clinical mutations can affect—secretase activity by different.pdf. 10.1111/j.1471-4159.2005.03578.x 16405513

[104] Copyright EN, Vol N. Presenilin mutations in familial alzheimer disease and transgenic mouse models accelerate neuronal lysosomal pathology. J Neuropathol Exp Neurol. 2004;63:821–30. 15330337 10.1093/jnen/63.8.821

[105] Lee J, Yu WH, Kumar A, Lee S, Mohan PS, Peterhoff CM, et al. Lysosomal proteolysis and autophagy require presenilin 1 and are disrupted by Alzheimer-related PS1 mutations. Cell. 2010;141:1146–58. 10.1016/j.cell.2010.05.008. 20541250 10.1016/j.cell.2010.05.008 PMC3647462

[106] Zhang X, Garbett K, Veeraraghavalu K, Wilburn B, Gilmore R, Mirnics K, et al. A role for presenilins in autophagy revisited: normal acidification of lysosomes in cells lacking PSEN1 and PSEN2. J Neurosci. 2012;32:8633–48. 10.1523/JNEUROSCI.0556-12.2012. 22723704 10.1523/JNEUROSCI.0556-12.2012 PMC3467018

[107] Coen K, Flannagan RS, Baron S, Carraro-lacroix LR, Wang D, Vermeire W, et al. Lysosomal calcium homeostasis defects, not proton pump defects, cause endo-lysosomal dysfunction in PSEN-deficient cells. Cell Biol. 2012;198:23–35. 10.1083/jcb.201201076. 10.1083/jcb.201201076 PMC3392942 22753898

[108] Goiran T, Alves C. Presenilins at the crossroad of a functional interplay between PARK2 / PARKIN and PINK1 to control mitophagy : implication for neurodegenerative diseases. Biol Psychiatry. 2017;13:2004–5. 10.1016/j.biopsych.2017.04.011. 10.1080/15548627.2017.1363950 PMC5788489 28914586

[109] Jonsson T, Stefansson H, Steinberg S, Jonsdottir I, Jonsson PV, Snaedal J, et al. Variant of *TREM2* associated with the risk of Alzheimer’s disease. N Engl J Med. 2013;368:107–16. 10.1056/NEJMoa1211103. 23150908 10.1056/NEJMoa1211103 PMC3677583

[110] Guerreiro R, Wojtas A, Bras J, Carrasquillo M, Rogaeva E, Majounie E, et al. *TREM2* variants in Alzheimer’s disease. N Engl J Med. 2013;368:117–27. 10.1056/NEJMoa1211851. 23150934 10.1056/NEJMoa1211851 PMC3631573

[111] Qin Q, Teng Z, Liu C, Li Q, Yin Y, Tang Y. TREM2, microglia, and Alzheimer’s disease. Mech Ageing Dev. 2021;195:111438. 10.1016/j.mad.2021.111438. 33516818 10.1016/j.mad.2021.111438

[112] Ulland TK, Song WM, Huang SC-C, Ulrich JD, Sergushichev A, Beatty WL, et al. TREM2 maintains microglial metabolic fitness in Alzheimer’s disease. Cell. 2017;170:649–63. 10.1016/j.cell.2017.07.023. 28802038 10.1016/j.cell.2017.07.023 PMC5573224

[113] Hu L, Liu J, Peng J, Li X, Huang Z, Zhang C, et al. TREM2 alleviates neuroinflammation by maintaining cellular metabolic homeostasis and mitophagy activity during early inflammation. Diseases. 2025;13:60. 39997067 10.3390/diseases13020060 PMC11854088

[114] Pang X, Chu Y, Zhou L, Chen M, You Y, Tang Y, et al. Trem2 deficiency attenuates microglial phagocytosis and autophagic-lysosomal activation in white matter hypoperfusion. J Neurochem. 2023;167:489–504. 10.1111/jnc.15987. 37823326 10.1111/jnc.15987

[115] Wang X, Xie Y, Fan X, Wu X, Wang D, Zhu L. Intermittent hypoxia training enhances Aβ endocytosis by plaque associated microglia via VPS35-dependent TREM2 recycling in murine Alzheimer’s disease. Alzheimer’s Res Ther. 2024;16:1–20. 38831312 10.1186/s13195-024-01489-6 PMC11145795

[116] Zhong L, Xu Y, Zhuo R, Wang T, Wang K, Huang R, et al. Soluble TREM2 ameliorates pathological phenotypes by modulating microglial functions in an Alzheimer’s disease model. Nat Commun. 2019;10:1365. 10.1038/s41467-019-09118-9. 30911003 10.1038/s41467-019-09118-9 PMC6433910

[117] Liang XH, Jackson S, Seaman M, Brown K, Kempkes B, Hibshoosh H, et al. Induction of autophagy and inhibition of tumorigenesis by beclin 1. Nature. 1999;402:672–6. 10.1038/45257. 10604474 10.1038/45257

[118] He C, Levine B. The beclin 1 interactome. Curr Opin Cell Biol. 2010;22:140–9. 10.1016/j.ceb.2010.01.001. 20097051 10.1016/j.ceb.2010.01.001 PMC2854269

[119] Erlich S, Alexandrovich A, Shohami E, Pinkas-Kramarski R. Rapamycin is a neuroprotective treatment for traumatic brain injury. Neurobiol Dis. 2007;26:86–93. 10.1016/j.nbd.2006.12.003. 17270455 10.1016/j.nbd.2006.12.003

[120] Diskin T, Tal-Or P, Erlich S, Mizrachy L, Alexandrovich A, Shohami E, et al. Closed head injury induces upregulation of Beclin 1 at the cortical site of injury. J Neurotrauma. 2005;22:750–62. 10.1089/neu.2005.22.750. 16004578 10.1089/neu.2005.22.750

[121] Pickford F, Masliah E, Britschgi M, Lucin K, Narasimhan R, Jaeger PA, et al. The autophagy-related protein beclin 1 shows reduced expression in early Alzheimer disease and regulates amyloid β accumulation in mice. J Clin Investig. 2008;118:JCI33585. 10.1172/JCI33585. 10.1172/JCI33585 PMC2391284 18497889

[122] Jaeger PA, Pickford F, Sun C-H, Lucin KM, Masliah E, Wyss-Coray T. Regulation of amyloid precursor protein processing by the Beclin 1 complex. PLoS ONE. 2010;5:e11102. 10.1371/journal.pone.0011102. 20559548 10.1371/journal.pone.0011102 PMC2886067

[123] Lee J-A, Gao F-B. Regulation of Aβ pathology by beclin 1: a protective role for autophagy? J Clin Investig. 2008;118:JCI35662. 10.1172/JCI35662. 10.1172/JCI35662 PMC2391068 18497881

[124] Saha A, Saleem S, Paidi RK, Biswas SC. BH3-only proteins Puma and Beclin1 regulate autophagic death in neurons in response to Amyloid-β. Cell Death Discov. 2021;7:356. 10.1038/s41420-021-00748-x. 34782612 10.1038/s41420-021-00748-x PMC8593071

[125] Guo R, Liu J, Min X, Zeng W, Shan B, Zhang M, et al. Reduction of DHHC5-mediated beclin 1 S-palmitoylation underlies autophagy decline in aging. Nat Struct Mol Biol. 2024;31:232–45. 10.1038/s41594-023-01163-9. 38177673 10.1038/s41594-023-01163-9

[126] Esteves AR, Filipe F, Magalhães JD, Silva DF, Cardoso SM. The role of Beclin-1 acetylation on autophagic flux in Alzheimer’s disease. Mol Neurobiol. 2019;56:5654–70. 10.1007/s12035-019-1483-8. 30661206 10.1007/s12035-019-1483-8

[127] Lucin KM, O’Brien CE, Bieri G, Czirr E, Mosher KI, Abbey RJ, et al. Microglial Beclin 1 regulates retromer trafficking and phagocytosis and is impaired in Alzheimer’s disease. Neuron. 2013;79:873–86. 10.1016/j.neuron.2013.06.046. 24012002 10.1016/j.neuron.2013.06.046 PMC3779465

[128] Xue Z, Zhang S, Huang L, He Y, Fang R, Fang Y. Upexpression of Beclin-1-dependent autophagy protects against beta-amyloid-induced cell injury in PC12 cells. J Mol Neurosci. 2013;51:180–6. 10.1007/s12031-013-9974-y. 23420039 10.1007/s12031-013-9974-y

[129] Chen J, Liang Y, Hu S, Jiang J, Zeng M, Luo M. Role of ATG7-dependent non-autophagic pathway in angiogenesis. Front Pharmacol. 2024;14:1266311. 10.3389/fphar.2023.1266311. 38269279 10.3389/fphar.2023.1266311 PMC10806190

[130] Collier JJ, Suomi F, Oláhová M, McWilliams TG, Taylor RW. Emerging roles of ATG7 in human health and disease. EMBO Mol Med. 2021;13:e14824. 10.15252/emmm.202114824. 34725936 10.15252/emmm.202114824 PMC8649875

[131] Inoue K, Rispoli J, Kaphzan H, Klann E, Chen EI, Kim J, et al. Macroautophagy deficiency mediates age-dependent neurodegeneration through a phospho-tau pathway. Mol Neurodegener. 2012;7:48. 10.1186/1750-1326-7-48. 22998728 10.1186/1750-1326-7-48 PMC3544596

[132] Cai Z, Wang S, Cao S, Chen Y, Penati S, Peng V, et al. Loss of ATG7 in microglia impairs UPR, triggers ferroptosis, and weakens amyloid pathology control. J Exp Med. 2025;222:e20230173. 10.1084/jem.20230173. 39945772 10.1084/jem.20230173 PMC11823820

[133] Pan H-Y, Valapala M. Regulation of autophagy by the glycogen synthase kinase-3 (GSK-3) signaling pathway. IJMS. 2022;23:1709. 10.3390/ijms23031709. 35163631 10.3390/ijms23031709 PMC8836041

[134] Avrahami L, Farfara D, Shaham-Kol M, Vassar R, Frenkel D, Eldar-Finkelman H. Inhibition of glycogen synthase kinase-3 ameliorates β-amyloid pathology and restores lysosomal acidification and mammalian target of rapamycin activity in the Alzheimer disease mouse model. J Biol Chem. 2013;288:1295–306. 10.1074/jbc.M112.409250. 23155049 10.1074/jbc.M112.409250 PMC3543013

[135] Parr C, Carzaniga R, Gentleman SM, Van Leuven F, Walter J, Sastre M. Glycogen synthase kinase 3 inhibition promotes lysosomal biogenesis and autophagic degradation of the amyloid-β precursor protein. Mol Cell Biol. 2012;32:4410–8. 10.1128/MCB.00930-12. 22927642 10.1128/MCB.00930-12 PMC3486153

[136] Hoshi M, Takashima A, Noguchi K, Murayama M, Sato M, Kondo S, et al. Regulation of mitochondrial pyruvate dehydrogenase activity by tau protein kinase I/glycogen synthase kinase 3beta in brain. Proc Natl Acad Sci USA. 1996;93:2719–23. 10.1073/pnas.93.7.2719. 8610107 10.1073/pnas.93.7.2719 PMC39697

[137] Rajan KB, Weuve J, Barnes LL, McAninch EA, Wilson RS, Evans DA. Population estimate of people with clinical Alzheimer’s disease and mild cognitive impairment in the United States (2020–2060). Alzheimer’s Dementia. 2021;17:1966–75. 10.1002/alz.12362. 34043283 10.1002/alz.12362 PMC9013315

[138] Lopez-Lee C, Torres ERS, Carling G, Gan L. Mechanisms of sex differences in Alzheimer’s disease. Neuron. 2024;112:1208–21. 10.1016/j.neuron.2024.01.024. 38402606 10.1016/j.neuron.2024.01.024 PMC11076015

[139] Costa AJ, Oliveira RB, Wachilewski P, Nishino MS, Bassani TB, Stilhano RS, et al. Membrane estrogen receptor ERα activation improves tau clearance via autophagy induction in a tauopathy cell model. Brain Res. 2022;1795:148079. 10.1016/j.brainres.2022.148079. 36088959 10.1016/j.brainres.2022.148079

[140] Congdon EE. Sex differences in autophagy contribute to female vulnerability in Alzheimer’s disease. Front Neurosci. 2018;12:372. 10.3389/fnins.2018.00372. 29988365 10.3389/fnins.2018.00372 PMC6023994

[141] Brinton RD, Yao J, Yin F, Mack WJ, Cadenas E. Perimenopause as a neurological transition state. Nat Rev Endocrinol. 2015;11:393–405. 10.1038/nrendo.2015.82. 26007613 10.1038/nrendo.2015.82 PMC9934205

[142] McCarthy M, Raval AP. The peri-menopause in a woman’s life: a systemic inflammatory phase that enables later neurodegenerative disease. J Neuroinflamm. 2020;17:317. 10.1186/s12974-020-01998-9. 10.1186/s12974-020-01998-9 PMC7585188 33097048

[143] Zhao L, Mao Z, Brinton RD. A select combination of clinically relevant phytoestrogens enhances estrogen receptor β-binding selectivity and neuroprotective activities in vitro and in vivo. Endocrinology. 2009;150:770–83. 10.1210/en.2008-0715. 18818291 10.1210/en.2008-0715

[144] Horstman AM, Dillon EL, Urban RJ, Sheffield-Moore M. The role of androgens and estrogens on healthy aging and longevity. J Gerontol A Biol Sci Med Sci. 2012;67:1140–52. 10.1093/gerona/gls068. 22451474 10.1093/gerona/gls068 PMC3636678

[145] Burger HG, Hale GE, Robertson DM, Dennerstein L. A review of hormonal changes during the menopausal transition: focus on findings from the Melbourne Women’s Midlife Health Project. Hum Reprod Update. 2007;13:559–65. 10.1093/humupd/dmm020. 17630397 10.1093/humupd/dmm020

[146] Xiong J, Kang SS, Wang Z, Liu X, Kuo T-C, Korkmaz F, et al. FSH blockade improves cognition in mice with Alzheimer’s disease. Nature. 2022;603:470–6. 10.1038/s41586-022-04463-0. 35236988 10.1038/s41586-022-04463-0 PMC9940301

[147] Greenfield JP, Leung LW, Cai D, Kaasik K, Gross RS, Rodriguez-Boulan E, et al. Estrogen lowers Alzheimer β-Amyloid generation by stimulating trans-golgi network vesicle biogenesis. J Biol Chem. 2002;277:12128–36. 10.1074/jbc.M110009200. 11823458 10.1074/jbc.M110009200

[148] Barnes LL, Wilson RS, Bienias JL, Schneider JA, Evans DA, Bennett DA. Sex differences in the clinical manifestations of alzheimer disease pathology. Arch Gen Psychiatry. 2005;62:685. 10.1001/archpsyc.62.6.685. 15939846 10.1001/archpsyc.62.6.685

[149] Wang Z-F, Pan Z-Y, Xu C-S, Li Z-Q. Activation of G-protein coupled estrogen receptor 1 improves early-onset cognitive impairment via PI3K/Akt pathway in rats with traumatic brain injury. Biochem Biophys Res Commun. 2017;482:948–53. 10.1016/j.bbrc.2016.11.138. 27908726 10.1016/j.bbrc.2016.11.138

[150] Altmann A, Tian L, Henderson VW, Greicius MD, Alzheimer’s Disease Neuroimaging Initiative Investigators. Sex modifies the APOE-related risk of developing Alzheimer disease. Ann Neurol. 2014;75:563–73. 10.1002/ana.24135. 24623176 10.1002/ana.24135 PMC4117990

[151] Christensen A, Pike CJ. *APOE* genotype affects metabolic and Alzheimer-related outcomes induced by western diet in female EFAD mice. FASEB J. 2019;33:4054–66. 10.1096/fj.201801756R. 30509127 10.1096/fj.201801756R PMC6404574

[152] Yan Y, Wang X, Chaput D, Shin M-K, Koh Y, Gan L, et al. X-linked ubiquitin-specific peptidase 11 increases tauopathy vulnerability in women. Cell. 2022;185:3913-3930.e19. 10.1016/j.cell.2022.09.002. 36198316 10.1016/j.cell.2022.09.002 PMC9588697

[153] Davis EJ, Broestl L, Abdulai-Saiku S, Worden K, Bonham LW, Miñones-Moyano E, et al. A second X chromosome contributes to resilience in a mouse model of Alzheimer’s disease. Sci Transl Med. 2020;12:eaaz5677. 10.1126/scitranslmed.aaz5677. 32848093 10.1126/scitranslmed.aaz5677 PMC8409261

[154] Chen X, McClusky R, Itoh Y, Reue K, Arnold AP. X and Y chromosome complement influence adiposity and metabolism in mice. Endocrinology. 2013;154:1092–104. 10.1210/en.2012-2098. 23397033 10.1210/en.2012-2098 PMC3578992

[155] Mangold CA, Wronowski B, Du M, Masser DR, Hadad N, Bixler GV, et al. Sexually divergent induction of microglial-associated neuroinflammation with hippocampal aging. J Neuroinflamm. 2017;14:141. 10.1186/s12974-017-0920-8. 10.1186/s12974-017-0920-8 PMC5521082 28732515

[156] Han J, Fan Y, Zhou K, Blomgren K, Harris RA. Uncovering sex differences of rodent microglia. J Neuroinflamm. 2021;18:74. 10.1186/s12974-021-02124-z. 10.1186/s12974-021-02124-z PMC7972194 33731174

[157] Zhao W, Hou Y, Zhang Q, Yu H, Meng M, Zhang H, et al. Estrogen receptor β exerts neuroprotective effects by fine-tuning mitochondrial homeostasis through NRF1/PGC-1α. Neurochem Int. 2023;171:105636. 10.1016/j.neuint.2023.105636. 10.1016/j.neuint.2023.105636 39491237

[158] Adlimoghaddam A, Fayazbakhsh F, Mohammadi M, Babaei Z, Behrooz AB, Tabasi F, et al. Sex and region-specific disruption of autophagy and mitophagy in Alzheimer’s disease: linking cellular dysfunction to cognitive decline. Neuroscience. 2024;11:204. 10.1101/2024.10.30.621097. 10.1038/s41420-025-02490-0 PMC12033262 40287423

[159] Basak B, Holzbaur ELF. Mitophagy in neurons: mechanisms regulating mitochondrial turnover and neuronal homeostasis. J Mol Biol. 2025;437:169161. 10.1016/j.jmb.2025.169161. 40268233 10.1016/j.jmb.2025.169161

[160] Veverova K, Katonova A, Horakova H, Angelucci F, Laczó J, Hort J, et al. Mitophagy biomarkers are changed in the continuum of Alzheimer’s disease. Alzheimers Dement. 2023;19:e079718. 10.1002/alz.079718.

[161] Ou-Yang P, Cai Z-Y, Zhang Z-H. Molecular regulation mechanism of microglial autophagy in the pathology of Alzheimer’s disease. Aging Dis. 2023;14:1166. 10.14336/AD.2023.0106. 37163443 10.14336/AD.2023.0106 PMC10389815

[162] Wu A-G, Zhou X-G, Qiao G, Yu L, Tang Y, Yan L, et al. Targeting microglial autophagic degradation in NLRP3 inflammasome-mediated neurodegenerative diseases. Ageing Res Rev. 2021;65:101202. 10.1016/j.arr.2020.101202. 33161129 10.1016/j.arr.2020.101202

[163] Castellazzi M, Patergnani S, Donadio M, Giorgi C, Bonora M, Bosi C, et al. Autophagy and mitophagy biomarkers are reduced in sera of patients with Alzheimer’s disease and mild cognitive impairment. Sci Rep. 2019;9:20009. 10.1038/s41598-019-56614-5. 31882960 10.1038/s41598-019-56614-5 PMC6934625

[164] Pradeepkiran JA, Hindle A, Kshirsagar S, Reddy PH. Are mitophagy enhancers therapeutic targets for Alzheimer’s disease? Biomed Pharmacother. 2022;149:112918. 10.1016/j.biopha.2022.112918. 35585708 10.1016/j.biopha.2022.112918 PMC9148418

[165] Zhang H, Zhang Y, Zhang J, Jia D. Exercise alleviates cardiovascular diseases by improving mitochondrial homeostasis. JAHA. 2024;13:e036555. 10.1161/JAHA.124.036555. 39291488 10.1161/JAHA.124.036555 PMC11681480

[166] Roberts FL, Markby GR. New insights into molecular mechanisms mediating adaptation to exercise; a review focusing on mitochondrial biogenesis, mitochondrial function, mitophagy and autophagy. Cells. 2021;10:2639. 10.3390/cells10102639. 34685618 10.3390/cells10102639 PMC8533934

[167] Fishbein AB, Knutson KL, Zee PC. Circadian disruption and human health. J Clin Investig. 2021;131:e148286. 10.1172/JCI148286. 34596053 10.1172/JCI148286 PMC8483747

[168] Di T, Zhou Z, Liu F, Chen Y, Wang L. Autophagy and circadian rhythms: interactions and clinical implications. Biocell. 2024;48:33–45. 10.32604/biocell.2023.031638.

[169] Ma D, Li S, Molusky MM, Lin JD. Circadian autophagy rhythm: A link between clock and metabolism? Trends Endocrinol Metab. 2012;23:319–25. 10.1016/j.tem.2012.03.004. 22520961 10.1016/j.tem.2012.03.004 PMC3389582

[170] Schmukler E, Pinkas-Kramarski R. Autophagy induction in the treatment of Alzheimer’s disease. Drug Dev Res. 2020;81:184–93. 10.1002/ddr.21605. 31782539 10.1002/ddr.21605

[171] Fernandes SM, Mayer J, Nilsson P, Shimozawa M. How close is autophagy-targeting therapy for Alzheimer’s disease to clinical use? A summary of autophagy modulators in clinical studies. Front Cell Dev Biol. 2025;12:1520949. 10.3389/fcell.2024.1520949. 39845082 10.3389/fcell.2024.1520949 PMC11750832

[172] Zhang W, Xu C, Sun J, Shen H-M, Wang J, Yang C. Impairment of the autophagy–lysosomal pathway in Alzheimer’s diseases: pathogenic mechanisms and therapeutic potential. Acta Pharm Sin B. 2022;12:1019–40. 10.1016/j.apsb.2022.01.008. 35530153 10.1016/j.apsb.2022.01.008 PMC9069408

[173] Salari A, Roghani M, Khalili M. HMG-CoA reductase inhibitor simvastatin ameliorates trimethyltin neurotoxicity and cognitive impairment through reversal of Alzheimer’s-associated markers. Metab Brain Dis. 2024;40:74. 10.1007/s11011-024-01515-4. 39704877 10.1007/s11011-024-01515-4

[174] Li Z-Y, Chen L-H, Zhao X-Y, Chen H, Sun Y-Y, Lu M-H, et al. Clemastine attenuates AD-like pathology in an AD model mouse via enhancing mTOR-mediated autophagy. Exp Neurol. 2021;342:113742. 10.1016/j.expneurol.2021.113742. 33965410 10.1016/j.expneurol.2021.113742

[175] La Barbera L, Vedele F, Nobili A, Krashia P, Spoleti E, Latagliata EC, et al. Nilotinib restores memory function by preventing dopaminergic neuron degeneration in a mouse model of Alzheimer’s disease. Prog Neurobiol. 2021;202:102031. 10.1016/j.pneurobio.2021.102031. 33684513 10.1016/j.pneurobio.2021.102031

[176] Subramanian A, Tamilanban T, Alsayari A, Ramachawolran G, Wong LS, Sekar M, et al. Trilateral association of autophagy, mTOR and Alzheimer’s disease: potential pathway in the development for Alzheimer’s disease therapy. Front Pharmacol. 2022;13:1094351. 10.3389/fphar.2022.1094351. 36618946 10.3389/fphar.2022.1094351 PMC9817151

[177] Sun C, Gao X, Sha S, Wang S, Shan Y, Li L, et al. Berberine alleviates Alzheimer’s disease by activating autophagy and inhibiting ferroptosis through the JNK-p38MAPK signaling pathway. Int Immunopharmacol. 2025;155:114550. 10.1016/j.intimp.2025.114550. 40215776 10.1016/j.intimp.2025.114550

[178] Bonavita R, Vitale F, Verdicchio LV, Williams SV, Caporaso MG, Fleming A, et al. Small HSPs at the crossroad between protein aggregation, autophagy and unconventional secretion: clinical implications and potential therapeutic opportunities in the context of neurodegenerative diseases. Front Cell Dev Biol. 2025;13:1538377. 10.3389/fcell.2025.1538377. 40385290 10.3389/fcell.2025.1538377 PMC12081433

[179] Puglisi-Allegra S, Lazzeri G, Busceti CL, Giorgi FS, Biagioni F, Fornai F. Lithium engages autophagy for neuroprotection and neuroplasticity: translational evidence for therapy. Neurosci Biobehav Rev. 2023;148:105148. 10.1016/j.neubiorev.2023.105148. 36996994 10.1016/j.neubiorev.2023.105148

[180] Gao L, Hu S, Lv Y, Zheng G, Lin Z. Overexpression of LINC00672 promotes autophagy in Alzheimer’s disease by upregulating GPNMB. PLoS ONE. 2025;20:e0322708. 10.1371/journal.pone.0322708. 40367036 10.1371/journal.pone.0322708 PMC12077738

[181] Chandra S, Jana M, Pahan K. Aspirin induces lysosomal biogenesis and attenuates amyloid plaque pathology in a mouse model of Alzheimer’s disease via PPARα. J Neurosci. 2018;38:6682–99. 10.1523/JNEUROSCI.0054-18.2018. 29967008 10.1523/JNEUROSCI.0054-18.2018 PMC6067079

[182] Libro R, Giacoppo S, SoundaraRajan T, Bramanti P, Mazzon E. Natural phytochemicals in the treatment and prevention of dementia: an overview. Molecules. 2016;21:518. 10.3390/molecules21040518. 27110749 10.3390/molecules21040518 PMC6274085

[183] Cui Z, Zhao X, Amevor FK, Du X, Wang Y, Li D, et al. Therapeutic application of quercetin in aging-related diseases: SIRT1 as a potential mechanism. Front Immunol. 2022;13:943321. 10.3389/fimmu.2022.943321. 35935939 10.3389/fimmu.2022.943321 PMC9355713

[184] Iyaswamy A. Fe65-engineered neuronal exosomes encapsulating corynoxine-B ameliorate cognition and pathology of Alzheimer’s disease. Signal Transduct Target Ther. 2023;8:404. 37867176 10.1038/s41392-023-01657-4 PMC10590775

[185] Cordero JG, García-Escudero R, Avila J, Gargini R, García-Escudero V. Benefit of oleuropein aglycone for Alzheimer’s disease by promoting autophagy. Oxid Med Cell Longev. 2018;2018:5010741. 10.1155/2018/5010741. 29675133 10.1155/2018/5010741 PMC5838478

[186] Shokouhi Asl AS, Sayahi MH, Hashempur MH, Irajie C, Alaeddini AH, Ghafouri SN, et al. Cinnamic acid conjugated with triazole acetamides as anti-Alzheimer and anti-melanogenesis candidates: an in vitro and in silico study. Sci Rep. 2025;15:655. 10.1038/s41598-024-83020-3. 39754023 10.1038/s41598-024-83020-3 PMC11698978

[187] Zhang W, Ding J, Wang L, Wu C, Mao J, Ding K, et al. Tomatidine ameliorates diabetes-induced cognitive impairment and tau hyperphosphorylation through the AMPK-TFEB pathway. J Neurochem. 2025;169:e70087. 10.1111/jnc.70087. 40387453 10.1111/jnc.70087

[188] Di Meco A, Li J-G, Blass BE, Abou-Gharbia M, Lauretti E, Praticò D. 12/15-lipoxygenase inhibition reverses cognitive impairment, brain amyloidosis, and tau pathology by stimulating autophagy in aged triple transgenic mice. Biol Psychiatry. 2017;81:92–100. 10.1016/j.biopsych.2016.05.023. 27499089 10.1016/j.biopsych.2016.05.023

[189] Hu L, Liu J, Peng J, Li X, Huang Z, Zhang C, et al. TREM2 alleviates neuroinflammation by maintaining cellular metabolic homeostasis and mitophagy activity during early inflammation. Diseases. 2025;13:60. 10.3390/diseases13020060. 39997067 10.3390/diseases13020060 PMC11854088

[190] Wang X, Xie Y, Fan X, Wu X, Wang D, Zhu L. Intermittent hypoxia training enhances Aβ endocytosis by plaque associated microglia via VPS35-dependent TREM2 recycling in murine Alzheimer’s disease. Alz Res Therapy. 2024;16:121. 10.1186/s13195-024-01489-6. 10.1186/s13195-024-01489-6 PMC11145795 38831312

[191] Lauretti E, Dincer O, Praticò D. Glycogen synthase kinase-3 signaling in Alzheimer’s disease. Biochim Biophys Acta BBA Mol Cell Res. 2020;1867:118664. 10.1016/j.bbamcr.2020.118664. 10.1016/j.bbamcr.2020.118664 PMC7047718 32006534

[192] Jiang R, Shimozawa M, Mayer J, Tambaro S, Kumar R, Abelein A, et al. Autophagy impairment in app knock-in Alzheimer’s model mice. Front Aging Neurosci. 2022;14:878303. 10.3389/fnagi.2022.878303. 35663567 10.3389/fnagi.2022.878303 PMC9160569

[193] Zhang D, Zhang Y, Pan J, Cao J, Sun X, Li X, et al. Degradation of NLRP3 by p62-dependent-autophagy improves cognitive function in Alzheimer’s disease by maintaining the phagocytic function of microglia. CNS Neurosci Ther. 2023;29:2826–42. 10.1111/cns.14219. 37072933 10.1111/cns.14219 PMC10493665

[194] Blaudin De Thé F-X, Lassus B, Schaler AW, Fowler SL, Goulbourne CN, Jeggo R, et al. P62 accumulates through neuroanatomical circuits in response to tauopathy propagation. Acta Neuropathol Commun. 2021;9:177. 10.1186/s40478-021-01280-w. 34727983 10.1186/s40478-021-01280-w PMC8561893

[195] Settembre C, Di Malta C, Polito VA, Arencibia MG, Vetrini F, Erdin S, et al. TFEB links autophagy to lysosomal biogenesis. Science. 2011;332:1429–33. 10.1126/science.1204592. 21617040 10.1126/science.1204592 PMC3638014

[196] Gu Z, Cao H, Zuo C, Huang Y, Miao J, Song Y, et al. TFEB in Alzheimer’s disease: from molecular mechanisms to therapeutic implications. Neurobiol Dis. 2022;173:105855. 10.1016/j.nbd.2022.105855. 36031168 10.1016/j.nbd.2022.105855

[197] Giong H-K, Hyeon SJ, Lee J-G, Cho H-J, Park U, Stein TD, et al. Tau accumulation is cleared by the induced expression of VCP via autophagy. Acta Neuropathol. 2024;148:46. 10.1007/s00401-024-02804-z. 39316141 10.1007/s00401-024-02804-z PMC11422276

[198] Mosconi L, Berti V, Dyke J, Schelbaum E, Jett S, Loughlin L, et al. Menopause impacts human brain structure, connectivity, energy metabolism, and amyloid-beta deposition. Sci Rep. 2021;11(1):10867. 10.1038/s41598-021-90084-y. 34108509 10.1038/s41598-021-90084-y PMC8190071

[199] Pinto-Almazán R, Calzada-Mendoza CC, Campos-Lara MG, Guerra-Araiza C. Effect of chronic administration of estradiol, progesterone, and tibolone on the expression and phosphorylation of glycogen synthase kinase-3β and the microtubule-associated protein tau in the hippocampus and cerebellum of female rat. J Neurosci Res. 2012;90:878–86. 10.1002/jnr.22808. 22183707 10.1002/jnr.22808

[200] Klinge CM. Estrogens regulate life and death in mitochondria. J Bioenergy Biomembr. 2017;49:307–24. 10.1007/s10863-017-9704-1. 10.1007/s10863-017-9704-1 28401437

[201] Shang D, Wang L, Klionsky DJ, Cheng H, Zhou R. Sex differences in autophagy-mediated diseases: toward precision medicine. Autophagy. 2021;17:1065–76. 10.1080/15548627.2020.1752511. 32264724 10.1080/15548627.2020.1752511 PMC8143224

[202] Congdon EE. Sex differences in autophagy contribute to female vulnerability in Alzheimer’s disease. Front Neurosci. 2018;12:372. 10.3389/fnins.2018.00372. 29988365 10.3389/fnins.2018.00372 PMC6023994

[203] Xue Y, Zuo S, Wang F, Qi X. From hormones to neurodegeneration: how FSH drives Alzheimer’s disease. Front Aging Neurosci. 2025;17:1578439. 10.3389/fnagi.2025.1578439. 40589620 10.3389/fnagi.2025.1578439 PMC12206753

[204] Caceres A, Jene A, Esko T, Perez-Jurado LA, Gonzalez JR. Extreme downregulation of chromosome Y and Alzheimer’s disease in men. Neurobiol Aging. 2020;90:150.e1-150.e4. 10.1016/j.neurobiolaging.2020.02.003. 32147245 10.1016/j.neurobiolaging.2020.02.003

[205] Wang L, Ma H, Huang P, Xie Y, Near D, Wang H, et al. Down-regulation of Beclin1 promotes direct cardiac reprogramming. Sci Transl Med. 2020;12:eaay7856. 10.1126/scitranslmed.aay7856. 33087505 10.1126/scitranslmed.aay7856 PMC8188650

[206] Guneykaya D, Ivanov A, Hernandez DP, Haage V, Wojtas B, Meyer N, et al. Transcriptional and translational differences of microglia from male and female brains. Cell Rep. 2018;24:2773-2783.e6. 10.1016/j.celrep.2018.08.001. 30184509 10.1016/j.celrep.2018.08.001

[207] Yanguas-Casás N, Crespo-Castrillo A, De Ceballos ML, Chowen JA, Azcoitia I, Arevalo MA, et al. Sex differences in the phagocytic and migratory activity of microglia and their impairment by palmitic acid. Glia. 2018;66:522–37. 10.1002/glia.23263. 29139169 10.1002/glia.23263

[208] Guillot-Sestier M-V, Araiz AR, Mela V, Gaban AS, O’Neill E, Joshi L, et al. Microglial metabolism is a pivotal factor in sexual dimorphism in Alzheimer’s disease. Commun Biol. 2021;4:711. 10.1038/s42003-021-02259-y. 34112929 10.1038/s42003-021-02259-y PMC8192523

[209] Keren-Shaul H, Spinrad A, Weiner A, Matcovitch-Natan O, Dvir-Szternfeld R, Ulland TK, et al. A unique microglia type associated with restricting development of Alzheimer’s disease. Cell. 2017;169:1276-1290.e17. 10.1016/j.cell.2017.05.018. 28602351 10.1016/j.cell.2017.05.018

[210] Klein SL, Flanagan KL. Sex differences in immune responses. Nat Rev Immunol. 2016;16:626–38. 10.1038/nri.2016.90. 27546235 10.1038/nri.2016.90

[211] Klinge CM. Estrogenic control of mitochondrial function. Redox Biol. 2020;31:101435. 10.1016/j.redox.2020.101435. 32001259 10.1016/j.redox.2020.101435 PMC7212490

[212] Park J, Shin H, Song H, Lim HJ. Autophagic regulation in steroid hormone-responsive systems. Steroids. 2016;115:177–81. 10.1016/j.steroids.2016.09.011. 27643453 10.1016/j.steroids.2016.09.011

[213] Fox SN, McMeekin LJ, Savage CH, Joyce KL, Boas SM, Simmons MS, et al. Estrogen-related receptor gamma regulates mitochondrial and synaptic genes and modulates vulnerability to synucleinopathy. NPJ Parkinsons Dis. 2022;8:106. 10.1038/s41531-022-00369-w. 35982091 10.1038/s41531-022-00369-w PMC9388660

[214] Demarest TG, Varma VR, Estrada D, Babbar M, Basu S, Mahajan UV, et al. Biological sex and DNA repair deficiency drive Alzheimer’s disease via systemic metabolic remodeling and brain mitochondrial dysfunction. Acta Neuropathol. 2020;140:25–47. 10.1007/s00401-020-02152-8. 32333098 10.1007/s00401-020-02152-8 PMC7537767

[215] Stephen TL, Cacciottolo M, Balu D, Morgan TE, LaDu MJ, Finch CE, et al. APOE genotype and sex affect microglial interactions with plaques in Alzheimer’s disease mice. Acta Neuropathol Commun. 2019;7:82. 10.1186/s40478-019-0729-z. 31113487 10.1186/s40478-019-0729-z PMC6528326

